# Antigen-dependent IL-12 signaling in CAR T cells promotes regional to systemic disease targeting

**DOI:** 10.1038/s41467-023-40115-1

**Published:** 2023-08-07

**Authors:** Eric Hee Jun Lee, John P. Murad, Lea Christian, Jackson Gibson, Yukiko Yamaguchi, Cody Cullen, Diana Gumber, Anthony K. Park, Cari Young, Isabel Monroy, Jason Yang, Lawrence A. Stern, Lauren N. Adkins, Gaurav Dhapola, Brenna Gittins, Wen-Chung Chang, Catalina Martinez, Yanghee Woo, Mihaela Cristea, Lorna Rodriguez-Rodriguez, Jun Ishihara, John K. Lee, Stephen J. Forman, Leo D. Wang, Saul J. Priceman

**Affiliations:** 1 Department of Hematology and Hematopoietic Cell Transplantation, City of Hope, Duarte, CA 91010 USA; 2 Irell and Manella Graduate School of Biological Sciences, Beckman Research Institute of City of Hope, Duarte, CA 91010 USA; 3 Department of Clinical and Translational Project Development, City of Hope, Duarte, CA 91010 USA; 4 Department of Surgery, City of Hope, Duarte, CA 91010 USA; 5 Department of Medical Oncology and Therapeutics Research, City of Hope, Duarte, CA 91010 USA; 6 Department of Bioengineering, Imperial College London, 86 Wood Lane, London, W120BZ UK; 7 Human Biology Division, Fred Hutchinson Cancer Center, Seattle, WA 98019 USA; 8 Department of Immuno-Oncology, Beckman Research Institute of City of Hope, Duarte, CA 91010 USA; 9 Department of Pediatrics, City of Hope, Duarte, CA 91010 USA

**Keywords:** Immunotherapy, Cell delivery, Interleukins, Translational immunology

## Abstract

Chimeric antigen receptor (CAR) T cell therapeutic responses are hampered by limited T cell trafficking, persistence, and durable anti-tumor activity in solid tumors. However, these challenges can be largely overcome by relatively unconstrained synthetic engineering strategies. Here, we describe CAR T cells targeting tumor-associated glycoprotein-72 (TAG72), utilizing the CD28 transmembrane domain upstream of the 4-1BB co-stimulatory domain as a driver of potent anti-tumor activity and IFNγ secretion. CAR T cell-mediated IFNγ production facilitated by IL-12 signaling is required for tumor cell killing, which is recapitulated by engineering an optimized membrane-bound IL-12 (mbIL12) molecule in CAR T cells. These T cells show improved antigen-dependent T cell proliferation and recursive tumor cell killing in vitro, with robust in vivo efficacy in human ovarian cancer xenograft models. Locoregional administration of mbIL12-engineered CAR T cells promotes durable anti-tumor responses against both regional and systemic disease in mice. Safety and efficacy of mbIL12-engineered CAR T cells is demonstrated using an immunocompetent mouse model, with beneficial effects on the immunosuppressive tumor microenvironment. Collectively, our study features a clinically-applicable strategy to improve the efficacy of locoregionally-delivered CAR T cells engineered with antigen-dependent immune-modulating cytokines in targeting regional and systemic disease.

## Abstract (web summary)

Targeting solid tumours by chimeric antigen receptor (CAR) T cells require strategies that improve trafficking and effector function of these cells in the immunologically hostile cancer microenvironment. Here, authors show that CAR T cells engineered with incorporation of the CD28 transmembrane domain to the 4-1BB costimulatory domain and a membrane-bound form of IL-12 can achieve efficient anti-tumour activity and promote systemic disease targeting via regional T cell delivery in multi-metastatic disease models.

## Introduction

Chimeric antigen receptor (CAR)-engineered T-cell therapies against solid tumors have largely failed to achieve the robust and durable responses observed in hematologic malignancies^1,2^. Several key distinctions exist between these malignancies, including both physical and immunological aspects, which may be driving the limited responses seen to date in solid tumor CAR T-cell clinical trials. These challenges have inspired next-generation strategies to optimize potent and selective CAR molecules, T-cell functionalities, routes of T-cell administration, and therapeutic combinations to improve solid tumor CAR T-cell therapies^3–6^.

Several key improvements to CAR molecules have been developed^4,7,8^. First, the antigen-binding domains, mainly single chain variable fragments (scFvs), have been affinity-tuned to selectively target low or high tumor antigen density^9,10^. These considerations factor in off-tumor antigen expression patterns that may cause unwanted safety issues. The extracellular spacer domain has been shown to regulate T-cell persistence through Fc-mediated clearance mechanisms, and its length and conformation can further impact the potency and selectivity of CAR T cells^11–13^. While studies have shown that varying transmembrane domains primarily regulate CAR expression stability, more recent evidence supports this domain in regulating CAR signaling^14,15^. It is widely supported that the co-stimulatory domain, typically CD28 or 4-1BB, can greatly impact CAR T-cell functionality and in vivo expansion and persistence for durable anti-tumor responses^16–18^. Identification of the optimal CAR molecule alone, though, has proven insufficient for producing durable responses in solid tumors and has called for CAR-extrinsic innovation to further augment therapeutic responses. Engineered immune-modulating cytokines or chimeric-switch receptors have been shown to enhance therapeutic activity, although these often unconditional strategies retain the risk of off-target signaling and toxicities and warrant further optimization^3,19–21^.

To address potential off-target toxicities and improve the immediate on-target activity of CAR T-cell therapies for solid tumors, local or regional routes of administration compared to systemic intravenous delivery are being explored. Locoregional delivery of T-cell therapies is an effective strategy to overcome limitations of cell trafficking, tissue distribution, and on-target off-tumor toxicities by localizing anti-tumor activity at sites of disease. We and others have demonstrated improved anti-tumor responses following locoregional intracerebroventricular delivery of CAR T cells for the treatment of recurrent adult glioblastoma, pediatric brain tumors, and brain metastasis and/or leptomeningeal disease^22–26^. Likewise, intrapleural administration of CAR T cells has been effective in targeting mesothelioma and other pleural diseases^27,28^. Direct local intratumoral administration has been utilized to test CAR T cells targeting tumor antigens with unknown safety profiles^29^. Regional intraperitoneal delivery of CAR T cells, by several groups including our own, has demonstrated improved anti-tumor activity against peritoneal metastasis compared with intravenous delivery^30,31^. However, each of these regional delivery approaches is accompanied with a potential risk of limited distribution outside of the regional space and thereby potential restricts targeting of systemic disease. Several groups have incorporated engineering approaches to improve persistence and trafficking of T cells^3,21,32^, which is likely paramount to targeting widespread metastatic disease following locoregional delivery of cell therapies.

In this study, we focus on expanding our development of tumor-associated glycoprotein-72 (TAG72)-directed CAR T cells for regional targeting of peritoneal cancer metastasis^31^. We empirically optimize our TAG72-CAR construct identifying that the CD28 transmembrane coupled with a 4-1BB co-stimulatory domain greatly improves T-cell functionality and yields durable complete responses in vivo. These studies now culminate in a phase 1 trial evaluating safety, feasibility, and bioactivity of TAG72-CAR T cells for the treatment of patients with advanced ovarian cancer (NCT05225363). Functional assessment reveal interferon gamma (IFNγ) as a key mediator of CAR T-cell cytotoxicity and recursive tumor cell killing in vitro, and further validate the role of IL-12 in potent IFNγ induction and downstream CAR T-cell activity. Additionally, we engineer a membrane-bound form of IL-12 (mbIL12) with an optimal transmembrane domain that improves potency of CAR T cells in vitro and in vivo. Mechanistically, mbIL12 cell surface expression and downstream signaling is antigen-dependent, requiring T-cell activation through CAR or T-cell receptor (TCR) to promote both *cis* and *trans* signaling. Further, we show that locoregionally-administered CAR T cells with antigen-dependent IL-12 signaling promotes durable control of regional disease and enhances peripheral CAR T-cell expansion and persistence that eradicate systemic disease in two regional/systemic disease mouse models using two CAR targets. Finally, we demonstrate safety and efficacy of mbIL12-engineered CAR T cells compared with soluble IL-12 infusions using an immunocompetent mouse model, with mbIL12-dependent modulation of the immunosuppressive tumor microenvironment by CAR T cells. These data not only critically inform on our clinical development program, but also broadly support the use of antigen-dependent immune-modulating cytokines in promoting regional to systemic disease targeting with CAR T-cell therapies for multiple solid tumor disease indications.

## Results

### CD28 transmembrane in CARs containing a 4-1BB costimulatory domain enhances anti-tumor functionality in vitro

We previously generated and preclinically evaluated second-generation TAG72-specific CAR T cells containing a 4-1BB intracellular costimulatory domain, which demonstrated potent anti-tumor activity using human xenograft peritoneal ovarian tumor models^31^. The decision to redesign the CAR molecule for optimal functionality was based on our preclinical studies showing a lack of durable anti-tumor activity^31^ as well as early phase 1 data using first-generation TAG72-CAR T cells demonstrating anti-idiotype antibody production in patients that likely contributed to a lack of CAR T-cell persistence and therapeutic responses^33^. First, we re-assessed the antigen-binding single chain variable fragment (scFv) domain of our TAG72-CAR construct in attempt to minimize the potential for anti-CAR immunogenicity and improve T-cell persistence. We utilized two additional scFvs (v15, and v59-15: a fusion between v15 and v59) based on the original humanized CC49 scFv (IDEC) that through affinity maturation showed reduced potential for anti-idiotype immunogenicity^34,35^. Two of three scFvs exhibited similar high-binding affinities toward TAG72 antigen (IDEC, *K*_D_ = 33 ± 20 nM; v15, *K*_D_ = 35 ± 10 nM; v59-15, not determined). For all related in vitro and in vivo studies, we used human ovarian cancer cell lines that are TAG72-negative (OVCAR8) or are varying in cell surface expression levels of TAG72 (OVCAR3, OV90, and OVCAR8-sTn) (Fig. S1). Using the original CAR backbone we previously published^31^, TAG72-CAR T cells with the v15 scFv demonstrated superior anti-tumor activity, both in vitro and in vivo, as compared with IDEC and v59-15 (Fig. S2).

**Fig. 1:**
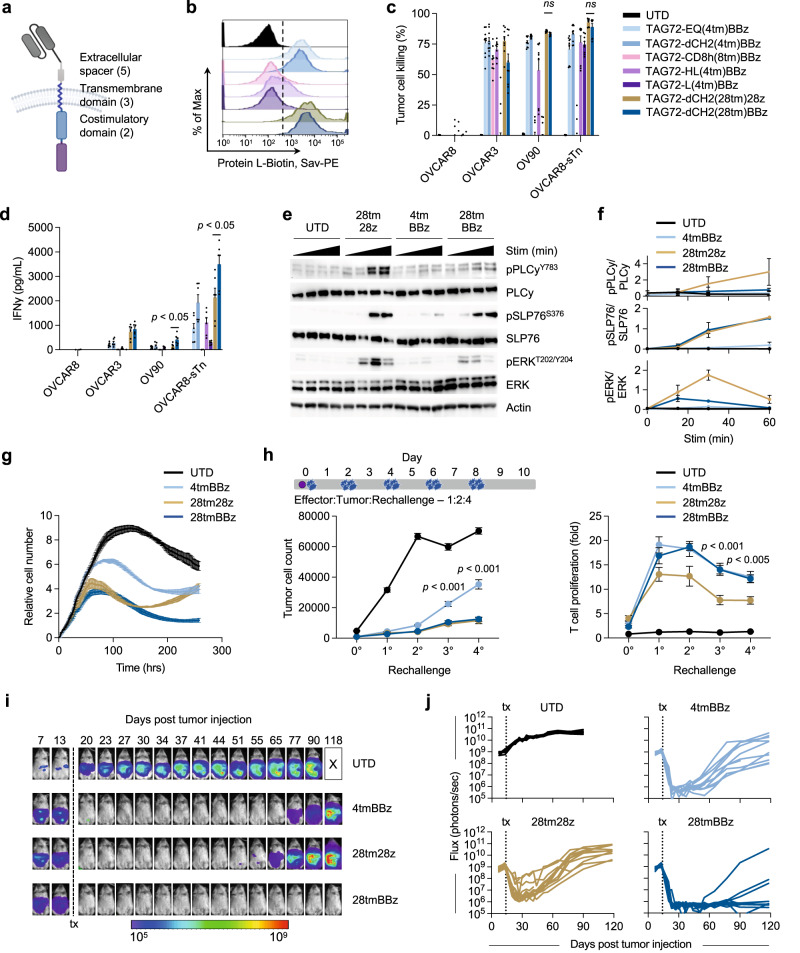
CD28 transmembrane in CARs containing a 4-1BB costimulatory domain enhances anti-tumor activity in vitro and in vivo. **a** Illustration of a TAG72-CAR T-cell containing the humanized scFv targeting TAG72 with varying five extracellular spacer domains (EQ, dCH2, CD8h, HL, L), three transmembrane domains (CD4tm, CD8tm, CD28tm), and two intracellular costimulatory domains (4-1BB, CD28) followed by a cytolytic domain (CD3z). **b** Untransduced (UTD) and seven different TAG72-CAR T cells positively enriched for CD19t were evaluated by flow cytometry for Protein L to detect the scFv. **c**, **d** In vitro tumor cell killing activity relative to UTD (**c**) and IFNγ production by ELISA (**d**), of CAR T cells against tumor targets (TAG72- OVCAR8; TAG72+ OVCAR3, OV90, and OVCAR8-sTn) after 24 hr (for ELISA) or 72 hr of co-culture at an effector:target (E:T) ratio of 1:4. *n* = 9 from three independent experiments. Data are presented as mean values ±SEM. *P* values indicate differences between TAG72-dCH2(28tm)28z and TAG72-dCH2(28tm)BBz using a two-tailed Student’s *t* test. **e** Western blotting analysis of early downstream signaling mediators following CAR T-cell stimulation of indicated TAG72-CAR T cells. **f** Quantification of band density of phosphoprotein over their respective total protein levels. *n* = 2 per timepoint, representative of two independent experiments. Data are presented as mean values ±SD. **g** TAG72-CAR T-cell killing of OV90 cells measured by xCELLigence over 10 days (E:T = 1:20). **h** Schema of repetitive tumor cell challenge assay (top). TAG72-CAR T cells were co-cultured with OV90 cells (E:T = 1:2) and rechallenged with OV90 cells every two days. Remaining viable tumor cells and fold change in TAG72-CAR T cells were quantified as described in Methods prior to each tumor cell rechallenge. n = 6–9/group from at least two independent experiments. Data are presented as mean values ±SEM. *P* values indicate differences between 28tm28z and 28tmBBz using a two-tailed Student’s *t* test. **i** Representative bioluminescent flux imaging of intraperitoneal (i.p.) OVCAR3(eGFP/ffluc) tumor-bearing female NSG mice treated i.p. with UTD or indicated TAG72-CAR T cells. **j** Quantification of flux (individual mice in each group) from treated OVCAR3 tumor-bearing mice. UTD (*n* = 8/group); TAG72-CAR T cells (*n* = 10/group). Curative responses: UTD: 0/10, 4tmBBz: 0/10, 28tm28z: 0/10, 28tmBBz: 4/10. *P* < 0.005 comparing 28tm28z and 28tmBBz using a Multiple Mann–Whitney test.

We next generated seven v15 scFv-based TAG72-CAR constructs with varying extracellular spacer domains and lengths (termed EQ, dCH2, CD8h, HL, and L), transmembrane domains (CD4tm, CD8tm, CD28tm), and intracellular costimulatory domains (CD28, 4-1BB) (Fig. 1a). While all seven CAR molecules comparably expressed the CD19t marker (Fig. S3, left), we observed higher cell surface CAR expression with Fc-derived spacers (EQ, dCH2) as measured by Protein L staining of the scFv (Fig. 1b) and anti-Fc antibodies to detect the extracellular spacer domain (Fig. S3, right). We next evaluated the cytotoxicity of these TAG72-CAR T cells using in vitro co-culture killing assays against cancer cell lines with varying TAG72 expression. In general, we found that TAG72-CAR T cells containing the dCH2 spacer domain showed superior functionality with the greatest tumor cell killing, highest CD137 activation, enhanced antigen-dependent T-cell proliferation, and robust IFNγ and IL-2 cytokine production (Figs. 1c, d, S4a, b, d). The three TAG72-CAR leads (dCH2(28tm)28z, dCH2(4tm)BBz, and dCH2(28tm)BBz) showed the highest T-cell activation and cytokine production. Additionally, we showed the greatest PD-1 exhaustive phenotype in CD28 costimulatory domain-containing CAR T cells (Fig. S4c), in line with previous reports using other CARs^16,36–38^. Interestingly, we showed the lowest PD-1 and highest IFNγ with TAG72-CAR T cells that contained the CD28tm and 4-1BB costimulatory domain. Some of these data were confirmed using HER2-CAR T cells, including showing the greatest tumor cell killing, highest CD137 activation, and enhanced antigen-dependent T-cell proliferation when CD28tm was coupled with the 4-1BB costimulatory domain (Fig. S4e–h). We also evaluated short-term signaling pathways following antigen stimulation in the three TAG72-CAR leads. While the 4-1BB costimulatory domain-containing CAR T cells demonstrated reduced downstream PLCy, SLP76, and ERK signaling as compared with CAR T cells containing the CD28 costimulatory domain, the addition of CD28tm to 4-1BB costimulation partially restored downstream signaling (Fig. 1e, f). These findings suggest that transmembrane domains of CARs have the potential to regulate early downstream signaling following CAR stimulation and can greatly impact CAR T-cell anti-tumor functionality.

### CD28 transmembrane domain in 4-1BB-based TAG72-CAR T cells induces potent therapeutic responses in vitro and in vivo

From these studies, we proceeded to further “stress-test” challenge the three TAG72-CAR T-cell lead candidates using an extended 10-day co-culture assay with OV90 tumor cells. TAG72-CAR T cells containing the CD28tm and 4-1BB costimulatory domain displayed the greatest anti-tumor activity (Fig. 1g). Recursive tumor cell killing assays showed two intriguing patterns; both 4-1BB costimulatory domain-containing CARs showed superior antigen-dependent T-cell expansion profiles, whereas the CD28 transmembrane domain-containing CAR T cells achieved better control of tumors over the rechallenge timepoints (Fig. 1h). Collectively, our in vitro studies identified three TAG72-CAR T-cell lead candidates with potent but varying anti-tumor functional profiles, which we selected for further assessment of their in vivo preclinical therapeutic activity.

Anti-tumor efficacy of these three TAG72-CAR leads was evaluated using previously established human peritoneal ovarian tumor xenograft models^31^. At day 14 following intraperitoneal (i.p.) OVCAR3 tumor injection, mice were treated with untransduced (UTD), TAG72-dCH2(4tm)BBz, TAG72-dCH2(28tm)28z, or TAG72-dCH2(28tm)BBz CAR T cells (5.0 × 10^6^) by regional i.p. delivery. Dramatic anti-tumor responses were shown with all three CAR T cells, which were sustained for up to 6 weeks in treated mice (Fig. 1i, j). While we observed tumor recurrences after 6–8 weeks post-treatment in mice treated with TAG72-dCH2(4tm)BBz or TAG72-dCH2(28tm)28z CAR T cells, TAG72-dCH2(28tm)BBz durably controlled tumors resulting in 4 out of 10 mice achieving complete therapeutic responses.

To better understand in vivo therapeutic differences amongst the three TAG72-CAR T-cell leads, we quantified CAR T cells in the peritoneal ascites and peripheral blood of mice during therapy. TAG72-dCH2(28tm)BBz CAR T cells showed the greatest proportion of cells in the ascites 14 days after CAR T-cell injection, compared to the other two CAR T cells (Fig. S5a, top). We additionally collected peritoneal ascites at the time of tumor recurrences (62 days post-treatment), and showed elimination of TAG72+ tumor cells only in TAG72-dCH2(28tm)BBz CAR T cell-treated mice (Fig. S5a bottom). We observed similar numbers of 4-1BB costimulatory domain-containing CAR T cells in the blood of mice at three timepoints, higher than CD28 costimulatory domain-containing CAR T cells (Fig. S5b). We also observed similar anti-tumor activity of the three CAR T-cell lead candidates using a second, more aggressive, human OV90 peritoneal ovarian tumor xenograft model (Fig. S6). These data largely matched patterns that were observed in our in vitro functional assays.

To address potential safety concerns of this optimized TAG72-CAR containing a new anti-TAG72 scFv and CAR backbone, we evaluated the on- and off-target normal cell killing potential of TAG72-dCH2(28tm)BBz CAR T cells. Little to no TAG72 expression was observed across 10 primary human normal cells evaluated by flow cytometry, with minimal CD137+ T-cell activation and cell killing by TAG72-CAR T cells, a finding that was consistent across five independent human CAR T-cell donors (Fig. S7). In sum, the TAG72-dCH2(28tm)BBz CAR construct showed the most optimal anti-tumor functionality in vitro with safe, potent, and durable anti-tumor efficacy in vivo.

### IL-12/IFNγ signaling regulates the anti-tumor activity of CAR T cells

**Fig. 2:**
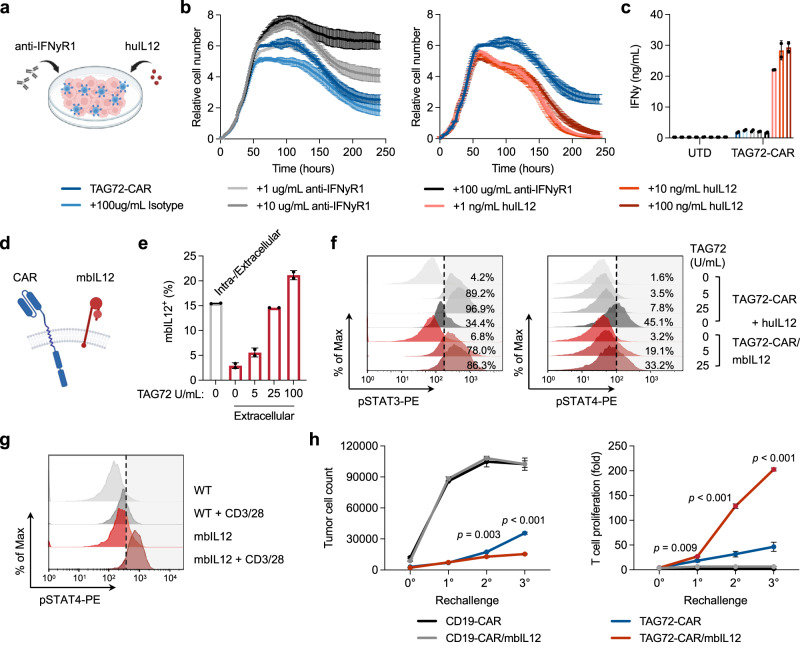
Membrane-bound IL-12 engineered TAG72-CAR T cells induce higher IFNγ, T-cell expansion, and anti-tumor activity in vitro. **a**–**c** Tumor cell killing of OV90 cells by TAG72-CAR T cells (E:T = 1:20) with the addition of varying concentrations of anti-IFNγR1 blocking antibody, isotype control, and recombinant human IL-12 cytokine measured by xCELLigence over 10 days (**a**, **b**). *n* = 2/group at each timepoint. Data are presented as mean values ±SD. At day 10, IFNγ levels in supernatants were quantified by ELISA (**c**). *n* = 2/group, representative of two independent experiments. Data are presented as mean values ±SD. **d** Illustration of TAG72-CAR/mbIL12 T cell. **e** Flow cytometric analysis of surface or intracellular expression of mbIL12 in TAG72-CAR T cells stimulated with varying concentrations of plate-bound TAG72. *n* = 2/group, representative of two independent experiments. Data are presented as mean values ±SD. **f** Intracellular flow cytometric analysis of phosphorylated STAT3 (pSTAT3, pY705) (left) and pSTAT4 (right) in TAG72-CAR and TAG72-CAR/mbIL12 T cells stimulated with varying concentrations of plate-bound TAG72 or recombinant huIL12 (10 ng/mL). **g** Intracellular flow cytometric analysis of pSTAT4 in TAG72-CAR T cells co-cultured with HT1080 (TAG72−) cells transduced with mbIL12. Cells were stimulated with Immunocult CD3/CD28 per manufacturer’s recommendation. Cells were gated on CAR T cells and evaluated for pSTAT4. **h** TAG72-CAR/mbIL12 T cells were co-cultured with OV90 cells (E:T = 1:3) and rechallenged with OV90 cells every 2 days. The remaining viable tumor cells and TAG72-CAR T-cell proliferation were quantified as described in Methods prior to each tumor cell rechallenge. *n* = 3/group, representative of two independent experiments. Data are presented as mean values ±SD. *P* values indicate differences between TAG72-CAR and TAG72-CAR/mbIL12 using a two-tailed Student’s *t* test.

Accumulating evidence supports IFNγ signaling in driving activity of CAR T cells^39–41^, and has recently been linked with CAR T-cell cytotoxicity^42^. Along with our observation that TAG72-dCH2(28tm)BBz CAR T cells secreted the highest levels of IFNγ in our studies, we hypothesized that IFNγ signaling contributed to the superior anti-tumor activity of TAG72-dCH2(28tm)BBz CAR T cells. To test this hypothesis, we inhibited IFNγ signaling using an anti-IFNγR1 blocking antibody or enhanced IFNγ secretion with a human recombinant interleukin-12 (huIL12) in our extended in vitro co-culture tumor cell killing assay (Fig. 2a). Strikingly, we saw dose-dependent dampening of tumor cell killing with blockade of IFNγ signaling using OV90 tumors cells (Fig. 2b, left) and OVCAR3 tumor cells (Fig. S8). We also observed dose-dependent enhancement of tumor cell killing with increasing concentrations of recombinant huIL12, which resulted in increased production of IFNγ by CAR T cells as determined by ELISA at the assay endpoint (Fig. 2b, right, and 2c). Interestingly, we observed only modest changes in T-cell proliferation following IFNγ blockade or the addition of huIL12 in this assay (Fig. S9). We further corroborated our findings using a recursive tumor cell killing assay, showing a requirement for IFNγ in sustained in vitro anti-tumor activity (Fig. S10). We sought to better understand the signaling kinetics downstream of IL-12 and in the context of CAR T-cell antigen stimulation. We first optimized an assay to confirm phosphorylated STAT4 (pSTAT4) downstream of huIL12 in TAG72-CAR T cells. Interestingly, we observed that while pSTAT4 levels peaked at 1 h and declined over the 24 h timecourse with recombinant huIL12 alone, pSTAT4 was sustained over the 24 h period in CAR T cells that were stimulated with plate-bound TAG72 antigen and huIL12 in combination (Fig. S11). These data suggest that IL-12 signaling enhanced CAR T-cell activation and cytotoxicity, and conversely CAR T-cell activation enhances persistent IL-12/pSTAT4 signaling.

### Engineered membrane-bound IL-12 signaling drives robust antigen-dependent CAR T cell-mediated tumor cell killing and T-cell proliferation

Building on our in vitro findings that IL-12-induced IFNγ signaling is critical for CAR T-cell anti-tumor activity, we next engineered synthetic IL-12 into our TAG72-CAR T cells. Due to the potential off-target toxicity induced by secreted IL-12^43^, we aimed to spatially restrict IL-12’s effect by building membrane-bound IL-12 (mbIL12) constructs with varying transmembrane domains (Fig. 2d). When we checked for their cell surface expression, we observed slightly enhanced expression of mbIL12(CD28tm) compared to mbIL12(B7.1tm), although both showed appreciable expression on TAG72-CAR T cells (Fig. S12a, b). We then evaluated the tumor cell killing activity of TAG72-CAR T cells expressing either of the two versions of mbIL12, and found that TAG72-CAR/mbIL12(CD28tm) T cells displayed greater tumor cell killing activity, T-cell expansion, and IFNγ production (Fig. S12c–e). In these in vitro assays, the T-cell cytotoxicity benefits with mbIL12 relied on IFNγ signaling (Fig. S13). Similar enhancement of activation and IFNγ production with mbIL12 was shown against human patient-derived TAG72+ gastric and ovarian cancer ascites cells, with little to no impact on the targeting of normal primary human cells (Fig. S14). T-cell functional benefits were also observed using HER2- and PSCA-targeting CAR T cells engineered with mbIL12(CD28tm) (Fig. S15a–h). mbIL12-engineered CAR T cells showed little/no changes in CD4/CD8 ratios, or markers of naive/memory phenotypes (CD62L, CCR7) in the in vitro functional assays (Fig. S15i-k).

We then compared the expression of cell surface mbIL12 on TAG72-CAR T cells that were rested in the absence of serum or exogenous cytokines prior to stimulation with plate-bound TAG72 antigen or control antigen. Without T-cell stimulation, cell-surface mbIL12 was detected at low levels on T cells, but was rather found largely intracellularly (Figs. 2e and S16a). We showed comparable mRNA transcripts of mbIL12 in stimulated and non-stimulated T cells, suggesting that low cell-surface mbIL12 expression was likely due to subcellular localization and not driven by differential EF1alpha promoter activity (Fig. S16b). Interestingly, significant dose-dependent increases in cell-surface expression of mbIL12 were observed in antigen-stimulated CAR T cells, in particular in CD137+-activated T-cell subsets (Figs. 2e and S16c).

Next, we interrogated downstream pSTAT4 expression in TAG72-CAR/mbIL12 T cells in response to CAR stimulation. We observed the expected phosphorylation of STAT3 in response to TAG72 antigen in both TAG72-CAR and TAG72-CAR/mbIL12 T cells, which was only slightly activated by huIL12. However, CAR stimulation showed dose-dependent increases in pSTAT4 in TAG72-CAR/mbIL12 T cells compared to TAG72-CAR T cells alone (Fig. 2f). We further evaluated the potential for *trans* signaling in TAG72-CAR/mbIL12 T cells. We transduced HT1080 (TAG72−) cells with mbIL12 and co-cultured them with T cells in the presence of soluble CD3/CD28 stimulation. Increased pSTAT4 in T cells was observed when cultured with both HT1080-mbIL12 and soluble CD3/CD28 and not with HT1080-mbIL12 alone or HT1080-WT with CD3/CD28 (Fig. 2g). Anti-tumor activity was further assessed in recursive tumor cell killing assays, in which we again observed enhanced tumor cell killing and CAR antigen-dependent T-cell expansion over multiple rechallenge timepoints in CAR T cells engineered with mbIL12 (Fig. 2h). Importantly, no expansion or survival benefits were observed in the absence of CAR antigen stimulation (Fig. S17). Collectively, these data suggest that mbIL12-engineered CAR T cells demonstrate improved in vitro anti-tumor activity and unexpectedly rely on CAR antigen stimulation, which we termed antigen-dependent IL-12 signaling.

### Superior anti-tumor activity by antigen-dependent IL-12 signaling in CAR T cells

**Fig. 3:**
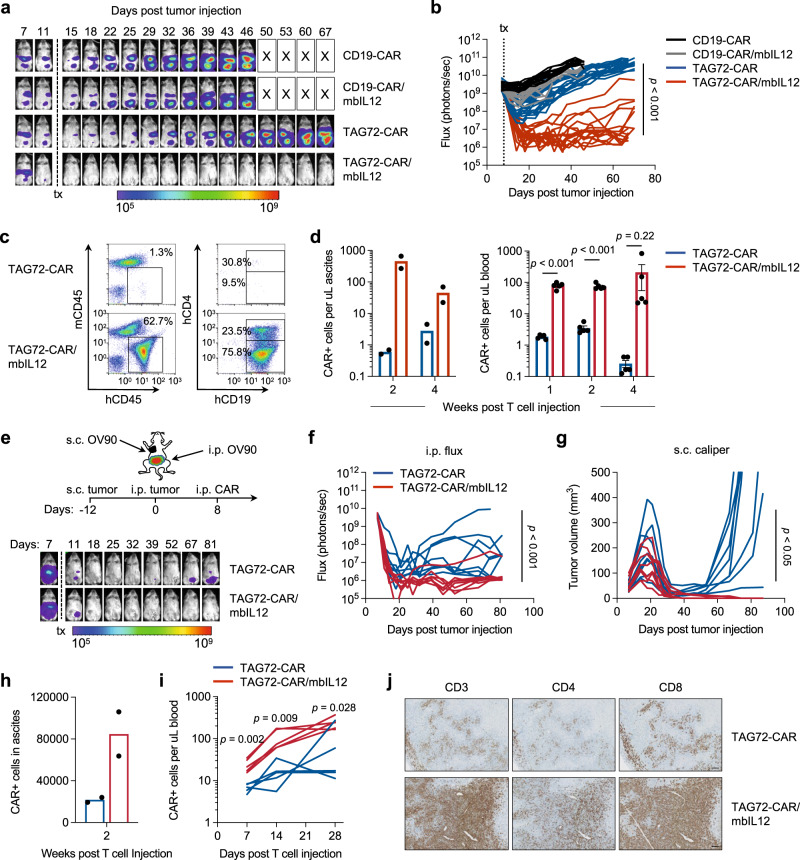
Locoregional intraperitoneal delivery of TAG72-CAR/mbIL12 T cells reduces tumor burden and increases regional and systemic CAR T-cell persistence in vivo. **a** Representative bioluminescent flux imaging of i.p. OV90(eGFP/ffluc) tumor-bearing mice treated i.p. with CD19-CAR, CD19-CAR/mbIL12, TAG72-CAR or TAG72-CAR/mbIL12 T cells. **b** Quantification of flux (individual mice per group) from OV90(eGFP/ffluc) tumor-bearing mice treated i.p. with CD19-CAR T cells (*n* = 12/group), CD19-CAR/mbIL12 T cells (*n* = 12/group), TAG72-CAR T cells (*n* = 20/group) and TAG72-CAR/mbIL12 T cells (*n* = 17/group). Combined data are from two independent studies. *P* value indicates differences between TAG72-CAR and TAG72-CAR/mbIL12 using a two-tailed Student’s *t* test. **c** Representative flow cytometric analysis of TAG72-CAR T cells per µL of peritoneal ascites. **d** Quantification of TAG72-CAR T cells per µL of peritoneal ascites (left) at weeks 2 and 4 post-treatment. *n* = 2/group per timepoint. Quantification of TAG72-CAR T cells per µL of peripheral blood (right) at weeks 1, 2, and 4 post-treatment. *n* = 5/group per timepoint. Data are presented as mean values ±SEM. *P* values indicate differences between TAG72-CAR and TAG72-CAR/mbIL12 using a two-tailed Student’s *t* test. **e** Schematic for subcutaneous (s.c.) and i.p. OV90 dual-tumor model and treatment (top). Representative bioluminescent flux imaging of dual-tumor-bearing mice treated i.p. with TAG72-CAR or TAG72-CAR/mbIL12 T cells (bottom). **f** Quantification of flux (individual mice per group) from mice treated i.p. with TAG72-CAR T cells (*n* = 8/group) and TAG72-CAR/mbIL12 T cells (*n* = 8/group). **g** Quantification of subcutaneous tumor volume (individual mice per group) from mice treated i.p. with TAG72-CAR T cells. **h** Quantification of TAG72-CAR T cells per µL of peritoneal ascites at week 2 post-treatment. *n* = 2/group. **i** Quantification of TAG72-CAR T cells per µL of peripheral blood at days 7, 14, 21, and 28 post-treatment. *n* = 5/group per timepoint. *P* values indicate differences between TAG72-CAR and TAG72-CAR/mbIL12 using a two-tailed Student’s *t* test. **j** Immunohistochemistry of CD3+ T cells in s.c. tumors at day 12 post-treatment. Data are representative of *n* = 3 mice/group. Scale bar = 100 µm.

We next evaluated the therapeutic potential of TAG72-CAR T cells with antigen-dependent IL-12 signaling. Using the aggressive i.p. OV90 tumor xenograft model, mice treated with TAG72-CAR/mbIL12 T cells showed durable anti-tumor responses as compared to TAG72-CAR T cells (Fig. 3a, b). Importantly, tumor-bearing mice treated with CD19-CAR T cells and CD19-CAR/mbIL12 T cells showed little differences in therapy, supporting the antigen-dependent nature of mbIL12. We observed a higher frequency of hCD45+ cells in the peritoneal ascites of mice treated with TAG72-CAR/mbIL12 T cells as compared to mice treated with TAG72-CAR T cells alone at 2 and 4 weeks post-treatment (Fig. 3c, d). Significantly higher and sustained levels of T cells were measured in the peripheral blood of mice treated with TAG72-CAR/mbIL12 T cells as compared with TAG72-CAR T cells alone (Fig. 3d, right). We replicated these findings using the i.p. OVCAR3 tumor xenograft model (Fig. S18).

### Improved systemic disease targeting by CAR T cells with antigen-dependent IL-12 signaling

One prevailing argument against the locoregional administration of CAR T cells is their potential spatial confinement, thereby preventing systemic therapy in patients with widespread metastatic disease^44^. However, our data suggest that regional intraperitoneally-administered mbIL12-engineered CAR T cells may have a greater capacity to target disease outside of the peritoneum. To test this, we established an OV90 tumor xenograft model with both regional i.p. metastasis and systemic s.c. tumors in the same mouse. OV90 (ffluc-negative) tumor cells were injected subcutaneously (s.c.) to track with calipers measurement and OV90 (eGFP/ffluc-expressing) tumor cells were i.p. injected and tracked with bioluminescent flux imaging (Fig. 3e, top). As we observed in previous experiments, i.p. anti-tumor responses were greater in mice regionally treated with TAG72-CAR/mbIL12 T cells as compared to TAG72-CAR T cells alone (Fig. 3e, f). While s.c. tumors initially regressed similarly in both treatment groups, all tumors recurred following TAG72-CAR T-cell treatment alone, whereas s.c. tumors were completely eradicated in all mice following TAG72-CAR/mbIL12 T-cell treatment (Fig. 3g). Again, higher levels of CAR T cells were observed in the peritoneal ascites and peripheral blood of mice treated with TAG72-CAR/mbIL12 T cells compared with CAR T cells alone (Fig. 3h, i). Immunohistochemistry (IHC) analysis of s.c. tumors at day 12 post-treatment demonstrated greater infiltration of CD4+ and CD8+ T cells as compared with TAG72-CAR T cells alone (Fig. 3j). Overall, these data support locoregional delivery of CAR T cells with engineered antigen-dependent IL-12 signaling in durable targeting of both regional and systemic disease.

**Fig. 4:**
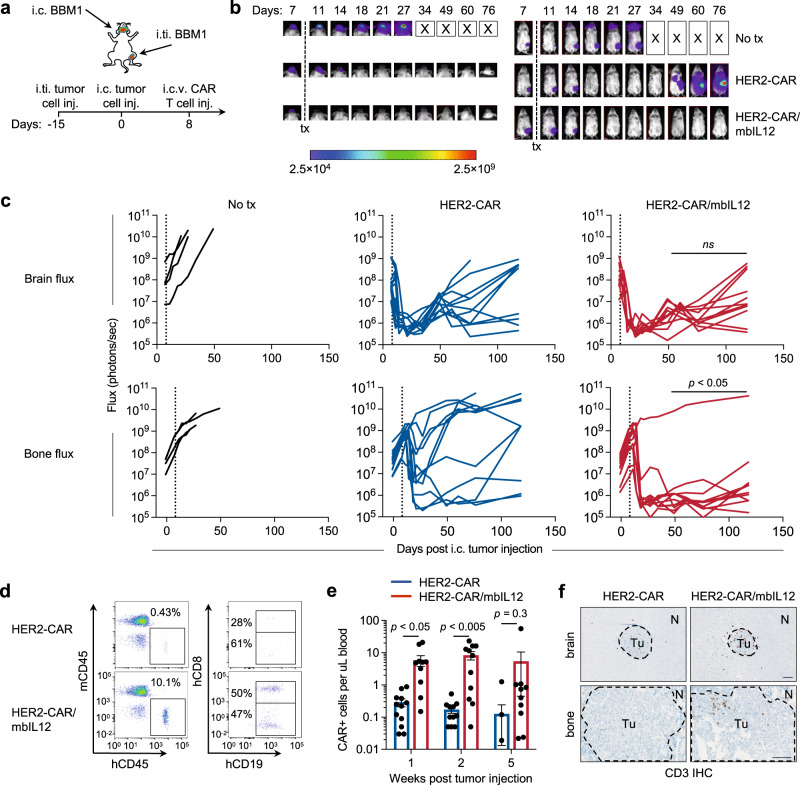
Locoregional intracerebroventricular delivery of HER2-CAR/mbIL12 T cells reduces tumor burden and increases regional and systemic CAR T-cell persistence in vivo. **a** Schematic for intratibial (i.ti.) and intracranial (i.c.) BBM1 dual-tumor model and treatment. **b** Representative bioluminescent flux imaging of dual-tumor-bearing mice left untreated (no tx), or treated by intracerebroventricular (i.c.v.) injection of HER2-CAR or HER2-CAR/mbIL12 T cells. **c** Quantification of brain (top) or bone (bottom) flux from individual mice treated i.c.v. with HER2-CAR T cells (*n* = 11/group), HER2-CAR/mbIL12 T cells (*n* = 11/group), or no tx (*n* = 4/group). *P* < 0.05 for bone flux, and not significant (*ns)* for brain flux, comparing HER2-CAR and HER2-CAR/mbIL12 using a Multiple Mann–Whitney test. **d**, **e** Representative flow cytometric analysis (**d**) and quantification (**e**) of HER2-CAR T cells per µL of blood at weeks 1, 2, and 5 post-treatment. **e** *n* = 11–12/group. Data are presented as mean values ±SEM. *P* values indicate differences between HER2-CAR and HER2-CAR/mbIL12 using a two-tailed Student’s *t* test. **f** Immunohistochemistry of CD3+ T cells in i.ti. and i.c. tumors at day 7 post-treatment. Data are representative of *n* = 3 mice/group. Scale bar = 100 µm.

To extend our finding that mbIL12 signaling enhances regional to systemic disease targeting by CAR T cells, we used the patient-derived HER2+ BBM1 tumor model we previously published in developing our HER2-CAR T cells^23^. Using this model system, we established a BBM1 tumor xenograft model with both regional intracranial (i.c.) brain metastasis and intratibial (i.ti.) bone metastasis in the same mouse. BBM1-ZsGreen-ffluc tumor cells were i.c. and i.ti. injected and tracked with bioluminescent flux imaging (Fig. 4a). Mice were treated with a single dose of HER2-CAR T cells by regional intracerebroventricular (i.c.v.) delivery corresponding to day 8 post i.c. tumor injection and day 23 post i.ti. tumor injection. We observed potent therapeutic responses in the brain using either HER2-CAR T cells alone or HER2-CAR T cells engineered with mbIL12 (Fig. 4b, c, top row). However, only mbIL12-engineered HER2-CAR T cells demonstrated curative responses in i.ti. bone metastases in the majority of treated mice, compared with heterogeneous responses with HER2-CAR T cells alone (Fig. 4b, c, bottom row). Similar to our observations in the dual-tumor ovarian cancer model above, we observed significant expansion and greater persistence of HER2-CAR/mbIL12 T cells in the peripheral blood of regional i.c.v. treated mice, as compared with HER2-CAR T cells alone (Fig. 4d, e), with persisting HER2-CAR/mbIL12 T cells in the blood showing a greater central memory phenotype (Fig. S19a, b), along with greater T-cell infiltration in both brain and bone metastases (Fig. 4f). Collectively, these two models strongly support the benefits of mbIL12 in targeting systemic disease following regional administration of CAR T cells.

### mbIL12-engineered CAR T cells demonstrate safety and efficacy in an immunocompetent mouse model of ovarian cancer peritoneal metastasis

The safety concerns of IL-12 have limited its therapeutic applications in humans to date. Infusion of soluble recombinant IL-12 in patients has resulted in unwanted toxicities, including colitis^45,46^. Additionally, engineering adoptive T-cell therapies with soluble IL-12, even under NFAT inducible promoter systems to limit IL-12 production to activated T cells has resulted in similar toxicities^43^. To test whether mbIL12-engineered CAR T cells are a safe therapeutic approach compared with soluble IL-12, we built a fully immunocompetent mouse model of TAG72+ ovarian cancer peritoneal metastasis, along with a murine version of our TAG72-CAR and mbIL12 constructs (Fig. S20a). TAG72-CAR T cells were efficiently manufactured as previously described using retrovirus transduction of murine splenic T cells^47^, and were further engineered to express mbIL12 (Fig. S20b, c). Murine ID8 ovarian cancer cells were stably transduced and cloned to express TAG72 using the mouse sialyl-transferace (mSTn) were used as tumor targets for our studies (Fig. S20d). Murine TAG72-CAR/mbIL12 T cells demonstrated increased activity and cytotoxicity against TAG72+ ID8 tumor cells, as compared with TAG72-CAR T cells alone (Fig. S20e–g). We observed a bias towards the antigen-dependent expansion of CD8+ CAR T cells, relative to the starting product, during the tumor cell-killing assays in vitro (Fig. S20c, h).

**Fig. 5:**
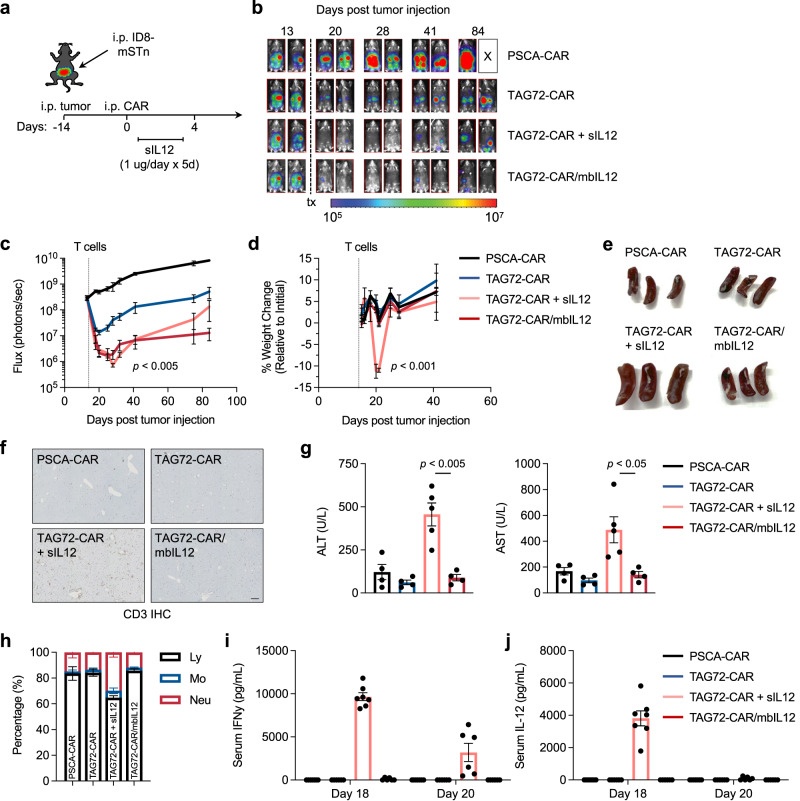
Locoregional intraperitoneal delivery of TAG72-CAR/mbIL12 T cells safely and effectively target ovarian cancer peritoneal metastasis in an immune-competent syngeneic mouse model. **a** Schematic for intraperitoneal (i.p.) ID8-mSTn tumor model and treatment. **b** Representative bioluminescence flux imaging of tumor-bearing mice, treated by intraperitoneal (i.p.) injection of indicated T cells. **c** Average tumor flux and **d** percent weight change in indicated T-cell treatments relative to pre-treatment weight (*n* = 5–7/group). *P* values indicate differences between TAG72-CAR and TAG72-CAR/mbIL12 in tumor flux using a two-tailed Student’s *t* test, and between TAG72-CAR/mbIL12 and TAG72-CAR + sIL12 in percent weight change at both days 20 and 21 using a two-tailed Student’s *t* test. **e** Spleen photographs (*n* = 3/group) and **f** representative CD3 IHC in livers harvested at 7 days post treatment. Quantification of CD3+ counts per mm^2^: PSCA-CAR: 37.7 ± 1.7, TAG72-CAR: 54.5 ± 7.6, TAG72-CAR + sIL12: 233.5 ± 19.2, TAG72-CAR/mbIL12: 68.3 ± 5.1. Scale bar = 200 µm. **g** Quantification of serum levels of ALT (left) and AST (right) from mice at 6 days post treatment. *n* = 4–5/group. *P* values indicate differences between TAG72-CAR/mbIL12 and TAG72-CAR + sIL12 using a two-tailed Student’s *t* test. **h** Percent lymphocytes, monocytes, and neutrophils from complete blood count analysis collected at 7 days post treatment. *n* = 4/group. **i**, **j** ELISA quantification of IFNγ (**i**) and IL-12 (**j**) cytokines in mouse serum at day 18 and 20 (day 4 and 6 post treatment, respectively) post tumor injection (*n* = 6–7/group). All data are presented as mean values ±SEM.

We performed a head-to-head safety and efficacy comparison of CAR T cells either engineered with mbIL12 or in combination with soluble IL-12 injections. TAG72+ ID8 (ffluc-positive) tumor cells were injected i.p. and tracked with bioluminescent flux imaging, and on day 14 post tumor injection were regionally i.p. treated with either TAG72-CAR T cells alone, TAG72-CAR/mbIL12 T cells, or TAG72-CAR T cells along with soluble IL-12 (sIL12) injections for 5 days (Fig. 5a). Potent therapeutic responses were seen in all treated mice, but were greater in mice regionally treated with TAG72-CAR/mbIL12 T cells or TAG72-CAR T cells with sIL12, as compared to TAG72-CAR T cells alone (Fig. 5b, c). Interestingly, at later timepoints, we observed recurrences in mice treated with CAR T cells and sIL12 injections, but durable tumor control in mice treated with CAR T cells engineered with mbIL12. While IL-12 benefited CAR T-cell therapy by injection or with T-cell engineering, we observed significant body weight loss in mice following TAG72-CAR T cells and sIL12 injections, and not in TAG72-CAR/mbIL12 treated mice (Fig. 5d). We confirmed systemic toxicities associated with sIL12 injections, with mice showing signs of splenomegaly (Figs. 5e and S21, top), signs of liver abnormalities and greater T-cell infiltration in the liver (Figs. 5f and S21, bottom), along with increased serum ALT and AST levels and changes in peripheral blood lymphocyte counts (Fig. 5g, h), while no appreciable systemic effects were observed with TAG72-CAR/mbIL12 T cells. Importantly, sIL12 injections resulted in increases in systemic IFNγ levels in the serum of TAG72-CAR T cell-treated mice, which was undetectable in TAG72-CAR/mbIL12 treated mice (Fig. 5i, j). These data support the safety and efficacy of regionally administered mbIL12-engineered CAR T cells in an immunocompetent mouse tumor model.

### mbIL12-engineered CAR T cells modify the immunosuppressive tumor microenvironment in peritoneal metastasis

**Fig. 6:**
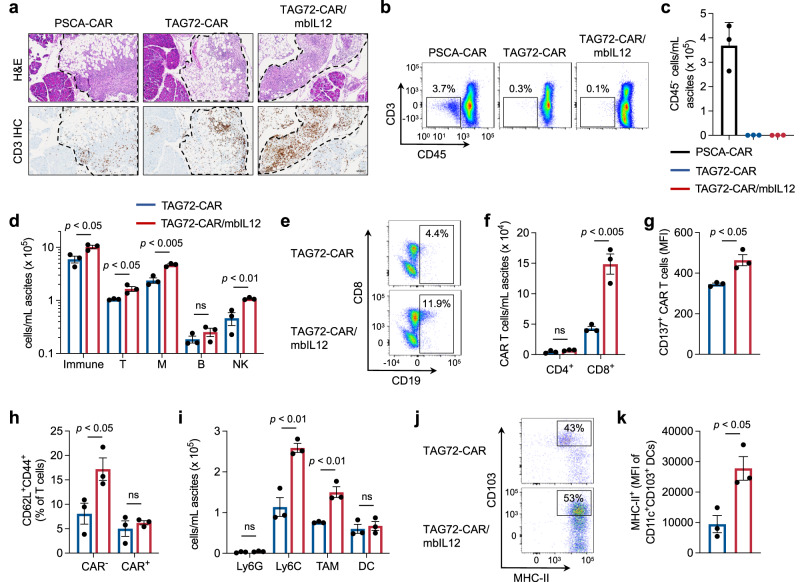
TAG72-CAR/mbIL12 T-cell therapy induces TME modifications in ovarian cancer peritoneal metastases in an immune-competent syngeneic mouse model. **a** Representative H&E and CD3 IHC in solid tumor masses collected from the upper omental region of i.p. ID8-mSTn tumor-bearing mice treated with indicated T cells. Scale bar = 100 µm. **b** Representative flow cytometric analysis of tumor cells (CD3-CD45- double negative) in peritoneal ascites. **c** Quantification of tumor cells (CD3− CD45− double negative) and **d** immune subsets (CD45+, CD3+, CD11b+, and NK+) as cells/mL in peritoneal ascites. **e** Representative flow cytometric analysis of percent CAR T cells (CD3+ CD19t+) and **f** quantification counts of CD4+ and CD8+ CAR T cells/mL in peritoneal ascites. **g** Quantification of mean fluorescent intensity (MFI) of CD137+ in CAR T cells in peritoneal ascites. **h** Quantification of percent CD62L+ CD44+ (Tcm) in both CAR+ and CAR− T cells in peritoneal ascites. **i** Quantification of myeloid cell counts (Ly6G+, Ly6C+, Ly6G−/C− double negative tumor-associated macrophages (TAM) and CD11c+ CD103+ dendritic cells (DC) as cells/mL in peritoneal ascites gated from total CD11b+ cells. Representative flow cytometric analysis of percent (**j**) and quantification of MFI (**k**) on CD103+ MHC Class II+ double positive DC in peritoneal ascites. All analyses represent data collected from ascites of ID8-mSTn tumor-bearing mice at 7 days post treatment, *n* = 3/group. All data are presented as mean values ±SEM. *P* values indicate differences between TAG72-CAR and TAG72-CAR/mbIL12 using a two-tailed Student’s *t* test.

IL-12 has numerous effects on the tumor microenvironment, including reshaping the myeloid cell compartment, promoting antigen presentation, and enhancing adoptive T-cell therapies^46,48–50^. We, therefore, assessed the impact of mbIL12-engineering of CAR T cells and its role in the tumor microenvironment in the ovarian cancer peritoneal metastasis model. Peritoneal tumors harvested from mice treated with TAG72-CAR/mbIL12 T cells showed increased T-cell infiltration as compared with TAG72-CAR T cells alone (Fig. 6a). At this early timepoint post therapy, both treated groups showed comparable potency in clearing tumor cells in the peritoneal ascites (Fig. 6b, c). We further evaluated the peritoneal ascites for changes in immune cell subsets by flow cytometry (Fig. S22), which showed increases in overall CD45+ immune cell counts, comprised of T cells, myeloid cells, and NK cells, with little change in total B cell counts (Figs. 6d and S23). TAG72-CAR T cells engineered with mbIL12 showed increased persistence of CD8+ T cells in the peritoneal ascites (Fig. 6e, f), and a small but significant increase in CD137 activation (Fig. 6g). Interestingly, while TAG72-CAR/mbIL12 T cells persisting showed little change in CD62L+ CD44+ naive/memory phenotypes, there was an increase in naive/memory phenotypes of non-CAR T cells in the peritoneal ascites (Fig. 6h). We then evaluated changes in the myeloid cell compartment in the peritoneal ascites, showing significant increases in total F4/80+ tumor-associated macrophages (TAM), along with increases in Ly6C+ inflammatory monocytes, without changes in Ly6G+ neutrophil and CD11c+ DC populations (Fig. 6i). However, we observed significant increases in MHC-II expression among the CD11c+ CD103+ DCs, suggesting an improved mature antigen-presenting phenotype. Collectively, these findings demonstrate beneficial modulation of the immunosuppressive tumor microenvironment in this model by mbIL12-engineered CAR T cells.

## Discussion

Our findings address major challenges associated with engineering safe and effective CAR T-cell strategies for solid tumors. Here, we fully optimized TAG72-specific CARs by varying the antigen-binding scFv, extracellular spacer, transmembrane, and intracellular costimulatory domains, and also validated the lead candidate using increasingly challenging in vitro studies and in vivo xenograft models of ovarian cancer. We are now underway in evaluating safety, feasibility, and bioactivity of regionally-delivered TAG72-CAR T cells in patients with advanced ovarian cancer in an open phase 1 trial (NCT05225363). Perhaps the most critical modification to the CAR was the incorporation of the CD28 transmembrane domain to the 4-1BB costimulatory domain, which allowed for durable anti-tumor activity and improved T-cell expansion in recursive tumor cell killing assays. Further, we identified IL-12-regulated IFNγ signaling in driving cytolytic activity and expansion/persistence of CAR T cells in vitro and in vivo. We showed that engineering a membrane-bound form of IL-12 enhanced CAR T-cell anti-tumor activity. Unexpectedly and perhaps most desirable, we observed that T-cell surface expression and signaling of our mbIL12 molecule was tightly controlled by antigen-dependent CAR activation.

Based on our own findings as well as emerging data in the CAR T-cell field, we reasoned that our clinical lead TAG72-CAR T cell’s superior performance against solid tumors is due to its ability to secrete high levels of IFNγ and enhance tumor cell killing. This notion was supported by demonstrating dampened TAG72-CAR T-cell activity with blockade of IFNγR1 signaling, which validated a recent study by Larson et al.^42^. Expanding on these findings, we now show enhanced CAR T-cell cytotoxicity with exogenous IL-12, a cytokine that canonically induces IFNγ secretion in T cells^30,51,52^. We further engineered CAR T cells targeting TAG72, HER2, or PSCA, with mbIL12 and showed similar improvements in anti-tumor activity. We demonstrated safety of our engineering approach compared to the toxicities associated with injected soluble IL-12, with beneficial effects of the immunosuppressive tumor microenvironment. Importantly, increased MHC class II expression in antigen-presenting cells along with modulation in phenotypes of other endogenous immune cells suggest that tumor control may also be enhanced by antigen spread. We anticipate that this approach also likely influences tumor vasculature through IFNγ signaling to further promote anti-tumor responses^30,41^. In support of these dynamic IL-12-driven effects, which warrants further investigation, we showed that mbIL12 signaling in T cells can also signal in *trans* with the capacity to impact other cell types within the solid tumor microenvironment. We believe these data have broad applicability to other CAR T-cell strategies and disease settings, and warrant further investigation.

Important to the CAR T-cell regional administration approach we used in this study and in the clinical design of our upcoming phase 1 trial (NCT05225363), we observed greater CAR T-cell persistence in the peritoneal ascites as well as in peripheral blood of treated mice with CAR T cells engineered with mbIL12. The durable persistence of TAG72-CAR T cells in the periphery led us to hypothesize that mbIL12 signaling in CAR T cells could solve one of the major hurdles facing local or regional delivery approaches, being the limited biodistribution and targeting of widespread metastatic disease. Indeed, our finding that regionally-delivered mbIL12-endowed CAR T cells better controlled regional as well as systemic disease compared to CAR T cells alone is a strong contender to resolving this issue. To further validate this phenomenon, we evaluated mbIL12-engineered HER2-CAR T cells, showing that intracerebroventricular delivery of CAR T cells effectively targeted both brain and extracranial bone metastases^23^. We, therefore, believe this to be a platform that will extend to other regional delivery approaches, including intrapleural, intralesional, and others.

Conditional or inducible gene engineering in CAR T cells is a major advancement in the field, with the potential to greatly improve the safety and overall efficacy of solid tumor-directed CAR T cells^3,53^. Various spatially and temporally activated gene promoters which drive the expression of CAR or other genes have been widely explored, ranging from heat-, light-, and hypoxia-inducible promoters, as well as synthetic Notch and drug-induced circuits^54–59^. While many of these approaches have not yet been investigated clinically, previous attempts to regulate gene expression by linking to conditionally-activated promoters, including the NFAT promoter^50^, have been clinically tested. Unfortunately, recent attempts at regulating the expression of a secretable IL-12 under an inducible NFAT promoter in an adoptively transferred T-cell therapy still resulted in unwanted toxicities due to systemic distribution of the cytokine^43^. To solve this issue, many groups have evaluated membrane-bound or -tethered approaches to limit the distribution of immune-modulating cytokines, including IL-12 and IL-15^60–63^. Here, we tethered the immune-modulating IL-12 to the surface of CAR T cells using an optimized CD28 transmembrane domain and discovered that its sustained cell surface expression and downstream signaling effects on CAR T cells were antigen-dependent. The precise mechanisms underlying this conditional mbIL12 signaling is under investigation by our group, and may be a viable approach to broadly regulate T-cell cytokine signaling including IL-2, IL-7, IL-15, and others.

In summary, this work highlights the advantages of comprehensively optimizing CAR functionality by varying different regions on the CAR molecule combined with systematically testing functional differences through challenging in vitro and in vivo preclinical modeling. We linked the enhancement of solid tumor killing by CAR T cells with the increased presence of IFNγ signaling, which we achieved by engineering antigen-dependent mbIL12 on CAR T cells. This addition not only improved regional tumor control but also enhanced systemic expansion and persistence of CAR T cells to promote the eradication of systemic disease in two model systems, which was a safe and effective strategy to promote IL-12-dependent functionalities. Our findings have the potential to broadly improve CAR T-cell therapeutic responses against multi-metastatic diseases.

## Methods

All studies were conducted in accordance with protocols approved by the City of Hope’s Internal Review Board (IRB) and Institutional Animal Care and Use Committee (IACUC). All relevant animal use guidelines and ethical regulations were followed. Further information on research design is available in the Reporting Summary linked to this article.

### Cell lines

The epithelial ovarian cancer line OVCAR3 (ATCC HTB-161) was cultured in RPMI-1640 (Lonza) containing 20% fetal bovine serum (FBS, Hyclone) and 1× antibiotic-antimycotic (1× AA, Gibco) (complete RPMI). The epithelial ovarian cancer line derived from metastatic ascites OV90 (CRL-11732) was cultured in a 1:1 mixture of MCDB 105 medium (Sigma) and Medium 199 (Thermo) adjusted to pH of 7.0 with sodium hydroxide (Sigma) and final 20% FBS and 1× AA. The epithelial ovarian cancer line OVCAR8 was a generous gift from Dr. Carlotta Glackin at the City of Hope and was cultured in complete RPMI-1640. The breast-to-brain metastasis patient-derived line BBM1 was cultured as previously described^64^. The mouse ovarian cancer cell line ID8 was cultured in Dulbecco’s Modified Eagles Medium (DMEM) supplemented with 10% FBS, 2 mM L-Glutamine (Fisher Scientific), and 25 mM HEPES (Irvine Scientific) (cDMEM). The human breast cancer cell line MDA-MB-468 (ATCC HTB-132) was engineered to express HER2 (Accession: NM_004448.4) under the control of the EF1α promotor via epHIV7 lentivirus transduction (468-HER2). All cells were cultured at 37 °C with 5% CO_2_. Human primary cell lines were obtained from Cell Biologics (Human Primary Colonic Epithelial Cells H-6047, Human Primary Esophageal Epithelial Cells H-6046, Human Primary Kidney Epithelial Cells H-6034, Human Primary Ovarian Epithelial Cells H-6036, Human Primary Pancreatic Epithelial Cells H-6037, Human Primary Proximal Tubular Epithelial Cells H-6015, Human Primary Small Intestine Epithelial Cells H-6051, Human Primary Stomach Epithelial Cells H-6039), Promocell (Human Cardiac Myocytes C-12810), and Lonza (Human Bronchial Epithelial Cells CC-2541) and cultured according to vendor’s specifications.

### DNA constructs, tumor lentiviral transduction, and retrovirus production

Human and murine tumor cells were engineered to express enhanced green fluorescent protein and firefly luciferase (eGFP/*ffluc*), or *ffluc* alone, by transduction with epHIV7 lentivirus carrying the eGFP/*ffluc* fusion or *ffluc* alone under the control of the EF1α promoter as described previously^23^. Murine ovarian cancer cell line ID8 was also engineered to express target antigen TAG72 via transduction with epHIV7 lentivirus carrying the murine *st6galnac-I* gene (mSTn) under the control of the EF1α. mSTn is the unique sialyltransferase responsible for generating surface expression of aberrant glycosylation sialyl-Tn (TAG72)^65^. The humanized scFv sequence used in the CAR construct was obtained from a monoclonal antibody clone huCC49 that targets TAG72^31^. The extracellular spacer domain included the 129-amino acid middle-length CH2-deleted version (ΔCH2) of the IgG4 Fc spacer^31^. The intracellular co-stimulatory signaling domain contained was a 4-1BB with a CD4 transmembrane domain. The CD3ζ cytolytic domain was previously described^31^. Variations in extracellular spacer domains, transmembrane domains, and intracellular co-stimulatory signaling domains were described previously^11,23,38^. The CAR sequence was separated from a truncated CD19 gene (CD19t) by a T2A ribosomal skip sequence, and cloned in an epHIV7 lentiviral backbone under the control of the EF1α promoter. The PSCA-BBζ CAR and HER2-BBζ CAR constructs were described previously^38^. The membrane-bound IL-12 (mbIL12) construct was generated using the p35 and p40 genes (p35, NC_000003.12; p40, NC_000005.10) separated by a G4S spacer, and linked to either the B7.1 or CD28 transmembrane domain. Lentivirus was generated as previously described^38^. Lentiviral titers were quantified using HT1080 cells based on CD19t or IL-12 cell surface expression using flow cytometry.

The scFv sequence from the mouse anti-human TAG72 antibody clone (CC49) was used to develop the murine CAR (mTAG72-CAR) construct. The extracellular spacer domain included the murine IgG1 region followed by a murine CD28 transmembrane domain^66,67^. The intracellular co-stimulatory signaling domain contained the murine 4-1BB followed by a murine CD3ζ cytolytic domain as previously described^68^. The CAR sequence was separated from a truncated murine CD19 gene (mCD19t) by a T2A ribosomal skip sequence. Murine membrane-bound IL-12 was generated using murine p40 and p35 subunits sequences linked to a cell membrane anchoring murine CD28 transmembrane domain sequence. All retrovirus constructs were cloned into the pMYs retrovirus backbone under the control of a hybrid MMLV/MSCV promoter (Cell Biolabs Inc). Production of retrovirus used to transduce primary murine T cells was performed as previously described^69^. Retrovirus was produced by transfecting the ecotropic retroviral packaging cell line, PLAT-E, with the addition of mTAG72-CAR retrovirus backbone plasmid DNA using FuGENE HD transfection reagent (Promega). Viral supernatants were collected after 24, 36, and 48 h, pooled, and stored at −80 °C in aliquots for future T-cell transductions. Control non-targeting murine mPSCA-CAR was generated as previously described^70^.

### T-cell Isolation, viral transduction, and ex vivo expansion

Leukapheresis products were obtained from consented research participants (healthy donors) under protocols approved by the City of Hope Internal Review Board (IRB), and enriched for T cells as previously described^38,71^. T-cell activation and transduction were performed as described previously^38^. Where indicated, we performed a second lentiviral transduction followed 24 hr after the first transduction. Cells were then ex vivo manufactured, enriched for CAR, and frozen as described previously^38^. Purity and cell surface phenotype of CAR T cells were analyzed by flow cytometry using antibodies and methods as described below.

For mouse T cells, splenocytes were obtained by manual digestion of spleens from female C57BL/6j mice. Enrichment of T cells was performed by EasySep™ mouse T-cell isolation kit per manufacturer’s protocol (StemCell Technologies). Single or dual retroviral transductions with mTAG72-CAR, mPSCA-CAR, and/or murine mbIL12 and subsequent expansion were performed as previously described, and cultured in cRPMI^69^.

### Flow cytometry

For flow cytometric analysis, cells were resuspended in FACS buffer (Hank’s balanced salt solution without Ca^2+^, Mg^2+^, or phenol red (HBSS^−/−^, Life Technologies) containing 2% FBS and 1 × AA). Cells were incubated with primary antibodies for 30 min at 4 °C in the dark. For secondary staining, cells were washed twice prior to 30 min incubation at 4 °C in the dark with either Brilliant Violet 510 (BV510), Brilliant Violet 570 (BV570), Brilliant Violet 605 (BV605), Brilliant Violet 650 (BV650), fluorescein isothiocyanate (FITC), phycoerythrin (PE), peridinin chlorophyll protein complex (PerCP), PerCP-Cy5.5, PECy7, allophycocyanin (APC), or APC-Cy7 (or APC-eFluor780), eFluor506, PE/Dazzl” “594, PerCP-eFluor 710, BD Horizo” “Red 718 (R718), Alexa Fluor 488 (AF488), PE-Cy5, -conjugated antibodies were used. Antibodies against CD3 (BD Biosciences, Cat: 563109, Clone: SK7), CD4 (BD Biosciences, Cat: 340443, Clone: SK3), CD8 (BD Biosciences, Cat: 347313, Clone: SK1), CD19 (BD Biosciences, Cat: 557835, Clone: SJ25C1), mouse CD45 (BioLegend, Cat: 103145, Clone: 30-F11), CD45 (BD Biosciences, Cat: 555484, Clone: 2D1), CD69 (BD Biosciences, Cat: 341652, Clone: L78), CD137 (BD Biosciences, Cat: 555956, Clone: 4B4-1), mouse CD137 (Thermofisher, Cat: 25-1371-82, Clone: 17B5), mouse NK1.1 (BioLegend, Cat: 108733, Clone: PK163), mouse PD-1 (Thermofisher, Cat: 69-9985-80, Clone: J43), mouse LAG3 (BioLegend, Cat: 125227, Clone: C9B7W), mouse TIM-3 (BioLegend, Cat: 119704, Clone: RMT3-23), mouse CD11b (BioLegend, Cat: 101237, Clone: M1/70), CD44 (BioLegend, Cat: 103010, Clone: IM7), CD62L (BioLegend, Cat: 104412, Clone: MEL-14), CD80 (BD Biosciences, Cat: 740130, Clone: 16-10A1), mouse I-A/I-E (MHC Class II) (Thermofisher, Cat: 64-5321-80, Clone: M5/114.15.2), mouse CD274 (PD-L1) (BioLegend, Cat: 124312, Clone: 10 F.962), Ly6C (BioLegend, Cat: 128029, Clone: HK1.4), mouse CD11c (BioLegend, Cat: 117316, Clone: N418), mouse Ly6G (Biolegend, Cat: 127623, Clone: 1A8), mouse CD103 (BioLegend, Cat: 121426, Clone: 2E7), mouse F4/80 (BioLegend, Cat: 123127, Clone: BM8), mouse IL-12/IL-23 p40 (Thermofisher, Cat: 12-7123-41, Clone:17.8), Ep-CAM/CD326 (BioLegend, Clone: 9C4) (Cat: 324208), human IL-12 p40/p70 (BD Biosciences, Cat: 554575, Clone: C11:5), biotinylated Protein L (GenScript USA, Cat: M00097) (25), TAG72 (Novus Biologicals), Clone, Cat: NBP2-33128, muCC49), Donkey Anti-Rabbit Ig (Invitrogen, Cat: A-31573), Goat Anti-Mouse Ig (BD Biosciences, Cat: 550589), and streptavidin (BD Biosciences, Cat: 349023, 1:20 dilution) were used. Cell viability was determined using 4′, 6-diamidino-2-phenylindole (DAPI, Sigma, Cat: D8417). Unless otherwise stated, antibodies were used at a dilution of 1:100. Refer to Table S1 for the full antibody list. Flow cytometry was performed on a MACSQuant Analyzer 10 or MACSQuant Analyzer 16 (Miltenyi Biotec), and the data were analyzed with FlowJo software (v10.8.1, TreeStar).

For intracellular flow cytometry, CAR T cells were thawed and rested in IL-2 (50 U/mL) & IL-15 (0.5 ng/mL) overnight at 1 × 10^6^ cells/mL. On the following day, CAR T cells were washed twice in 1 × PBS and suspended at 1 × 10^6^ cells/mL in media without serum or cytokines. 1 × 10^5^ cells were plated per well in a 96-well plate to rest overnight. The next day, cells were stimulated with either soluble cytokine [IL-2 (50 U/mL), IL-15 (0.5 ng/mL), IL-12 (10 ng/mL)] or transferred to a high-binding 96-well plate pre-coated with indicated amounts of control or TAG72 antigen (BioRad). Reagents and buffers for flow cytometry processing were pre-chilled on ice unless otherwise stated. Following antigen stimulation, cells were washed with FACS buffer (supplemented with 0.1% sodium azide) and then fixed in pre-warmed 1× BD Phosflow Lyse/Fix buffer (558049) at 37°C for 10 min. Cells were then washed with FACS buffer and if required, stained with the extracellular antibodies on ice for 30 min in the dark. Stained cells were washed and suspended in pre-chilled (-20^o^C) BD Perm Buffer III (558050) and kept on ice for 30 min. Following a wash, cells were suspended in human FC block (Miltenyi Biotec Inc., FLP3330, 1:50) and kept on ice for 30 min, washed and stained at dilution of 1:5 with intracellular antibodies: PE-pSTAT3 (BD Biosciences, Cat: 562072, pY705) or PE-pSTAT4 (BD Biosciences, Cat: 562073, pY693). Refer to Table S1 for the full antibody list. Data was acquired on a MACSQuant Analyzer 16 cytometer (Miltenyi) and analyzed with FlowJo v10.8.

### In vitro tumor killing and T-cell functional assays

For tumor cell killing assays, CAR T cells and tumor targets were co-cultured at indicated effector:tumor (E:T) ratios in complete X-VIVO (for human T-cell assays) or cRPMI (for murine T-cell assays) without cytokines in 96-well plates for the indicated timepoints and analyzed by flow cytometry as described above. Tumor cells were plated overnight prior to the addition of T cells. Tumor cell killing by CAR T cells was calculated by comparing CD45-negative DAPI-negative (viable) cell counts relative targets co-cultured with untransduced (UTD) T cells. For tumor cell challenge assays, TAG72-CAR T cells engineered with or without mbIL12 were co-cultured with OV90 cells at 1:2 E:T ratio and rechallenged with OV90 cells every 2 days for up to five times. Similarly, HER2-CAR T cells engineered with or without mbIL12 were co-cultured with 468-HER2 tumor cells at 1:10 E:T ratio and rechallenged with 468-HER2 cells every three days. The remaining viable tumor cells and T cells were quantified by flow cytometry prior to every rechallenge and two or three days after the last rechallenge with tumor cells. For xCELLigence tumor cell killing assays, CAR T cells and tumor targets were co-cultured at indicated effector:tumor (E:T) ratios in complete X-VIVO without cytokines in 96-well plates for up to 10 days and analyzed by flow cytometry as described above.

To evaluate CAR T-cell activity against normal tissue, normal tissue cells were co-cultured with CAR T cells at indicated E:T ratios. Patient-derived primary gastric cancer ascites (GAS1) and ovarian cancer ascites (OAS3 and OAS4) were thawed immediately and evaluated in T-cell functional assays. After 48 h, CD137 expression and cell killing was evaluated by flow cytometry, and the supernatant was collected to quantify IFNγ by ELISA.

For T-cell activation assays, CAR T cells and tumor targets were co-cultured at the indicated E:T ratios in complete X-VIVO without cytokines in 96-well plates for the indicated timepoints and analyzed by flow cytometry for indicated markers of T-cell activation. For T-cell activation assays on plate-bound antigen, purified soluble TAG72 antigen (BioRad) was plated in duplicate at indicated TAG72 units overnight at 4 °C in 1× PBS in 96-well flat bottom high-affinity plates (Corning). Using a Bradford protein assay, the 20,000 units/mL stock solution of soluble TAG72 antigen was determined to be ~1.234 mg/mL of total protein. A designated number of TAG72-CAR T cells were then added in a fixed volume of 100 µL to each well and incubated for indicated times prior to the collection of cells for analysis of activation markers (CD69, CD137) by flow cytometry. Supernatants were also collected for analysis of cytokine production. For T-cell survival assays, T cells were plated at 1 × 10^6^ cells/mL in X-VIVO 10% FBS with or without cytokines and counted every two days. Cell concentration was adjusted to 1 × 10^6^ cells/mL with fresh media following each count day.

### ELISA cytokine assays

Supernatants from tumor cell killing assays or CAR T-cell activation assays on plate-bound TAG72 antigen were collected at indicated times and frozen at −20 °C for further use. Supernatants were then analyzed for secreted human IFNγ and IL-2 according to the Human IFNγ and IL-2 ELISA Ready-SET-GO!®; ELISA kit manufacturer’s protocol, respectively. Plates were read at 450 nm using a Wallac Victor3 1420 Counter (Perkin-Elmer) and the Wallac 1420 Workstation software. Mouse serum cytokine levels of murine IFNγ and IL-12 were measured after pre-dilution in sample diluent using murine IFNγ and IL-12 ELISA Ready-SET-GO!® ELISA kit (Invitrogen) kits according to manufacturer’s protocol, respectively. Plates were read at 450 nm using a Cytation3 imaging reader with Gen5 microplate software v3.05 (BioTek).

### Western blotting analysis

Cell pellets were thawed on ice. After thaw, cell pellets were resuspended in RIPA buffer consisting of 25 mM Tris-HCl (pH 8.5), 150 mM NaCl, 1 mM EDTA (pH 8.0), 1%(v/v) NP-40 substitute, 0.5%(w/v) Sodium Deoxycholate, 0.1%(w/v) SDS, 10 mM NaF, 1 mM NaOV, 10 mM β-glycerophosphate, and 1× of Halt Protease and Phosphatase Inhibitor Cocktail (Thermo Scientific). Lysates were incubated on ice for 30 min and then centrifuged at 17,200 × *g* for 20 min at 4 °C. Lysate supernatant was transferred to a new tube and analyzed for total protein concentration by Bradford protein assay. Laemmli sample buffer (BioRad) containing DTT (Sigma Aldrich) was added to proportional quantities of total protein and samples were heated at 95 °C for 5 min. Protein was separated on a 7.5% Criterion TGX Precast Midi Protein Gel (BioRad) using the Criterion Cell (BioRad) and transferred to 0.2um nitrocellulose blotting membrane (Genesee) in Tris-Glycine Transfer Buffer (Thermo Scientific) using the Trans-Blot Turbo Electrophoretic Transfer Cell (BioRad). Membranes were washed in deionized water, incubated in Ponceau S solution (Sigma Aldrich) to confirm protein transfer, and then washed in Tris-buffered saline containing 0.05% Tween20 (Sigma Aldrich) (TBST) for 1 min. Membranes were then blocked for 1 h at room temperature in a blocking buffer containing 5% PhosphoBLOCKER blocking reagent (Cell Biolabs) in TBST. After blocking, membranes were transferred to a blocking buffer containing primary antibodies and incubated overnight at 4 °C. All primary antibodies were sourced from Cell Signaling Technology and included actin (Cat: 3700, 1:2000), p44/42 MAPK (ERK1/2) (Cat: 4695, 1:1000), pp44/42 MAPK (pERK1/2) (Cat: 4370, 1:1000), SLP76 (Cat: 4958, 1:1000), pSLP76 (Cat: 14745, 1:1000), PLCγ1 (Cat: 5690, 1:1000), and pPLCγ1 (Cat: 14008, 1:1000). Membranes were washed in TBST and then incubated for 45 min at room temperature in blocking buffer containing either anti-rabbit or anti-mouse HRP-linked secondary antibody (Cell Signaling Technology, Cat: 7074 or 7076, 1:1000). Refer to Table S1 for full antibody list. Membranes were washed in TBST and imaged on the ChemiDoc Imaging System using SuperSignal chemiluminescent substrate (Thermo Scientific).

### In vivo studies

All animal experiments were performed under protocols approved by the City of Hope Institutional Animal Care and Use Committee. All mice were co-housed in a maximum barrier, pathogen- and opportunist-free animal facility. For in vivo intraperitoneal (i.p.) tumor studies, OVCAR3, OV90, or ID8 cells (5.0 × 10^6^) were prepared in a final volume of 500 µL HBSS^−/−^ and engrafted in >6 weeks old female NSG (Stock No: 005557) or C57BL/6j (Stock No: 000664) mice by i.p. injection. For subcutaneous (s.c.) tumor studies, OV90 cells (5 × 10^6^) were prepared in a final volume of 100 µL HBSS^−/−^ and injected under the skin of the abdomen of 6–8 weeks old female NSG mice. For in vivo intracranial (i.c.) tumor studies, BBM1 cells (0.2 × 10^6^) were prepared and injected as previously described^23^. For intratibial (i.ti.) tumor studies, BBM1 cells (0.2 × 10^6^) were prepared in a a final volume of 30 µL HBSS^−/−^ and injected in the tibia. Tumor growth was monitored at least once a week via non-invasive bioluminescence imaging (Xenogen, LagoX) and flux signals were analyzed with Aura software v.4.0 (Spectral Instruments Imaging). For imaging, mice were i.p. injected with 150 uL D-luciferin potassium salt (Perkin-Elmer) suspended in PBS at 4.29 mg/mouse. At day 8 for OV90 and day 14 for OVCAR3 and ID8, mice were i.p. treated with indicated T cells (5 × 10^6^) in 500 uL final volume. At day 8 for BBM1 (brain) and day 23 for BBM1 (bone), mice were treated by intracerebroventricular (i.c.v.) injection with indicated T cells (0.5 × 10^6^) in 3 µL final volume^23^. Humane endpoints were used in determining survival. Mice were euthanized by CO_2_ and cervical dislocation upon signs of distress, such as a distended belly due to peritoneal ascites, labored or difficulty breathing, apparent weight loss, impaired mobility, or evidence of being moribund. At pre-determined time points or at moribund status, mice were euthanized and tissues and/or peritoneal ascites were harvested and processed for flow cytometry and immunohistochemistry as described above.

Peripheral blood was collected from isoflurane-anesthetized mice by retro-orbital (RO) bleed through heparinized capillary tubes (Chase Scientific) into polystyrene tubes containing a heparin/PBS solution (1000 units/mL, Sagent Pharmaceuticals). The total volume of each RO blood draw (~120 µL/mouse) was recorded. Red blood cells (RBCs) were lysed with 1× Red Cell Lysis Buffer (Sigma) according to the manufacturer’s protocol and then washed, stained, and analyzed by flow cytometry as described above. Cells from peritoneal ascites were collected from euthanized mice by injecting 2 mL cold 1× PBS into the i.p. cavity, which was drawn up via syringe and stored on ice until further processing. In some studies, cell-free ascites fluid was also collected and stored at −80 °C for future use. RBC-depleted ascites cells were washed, stained, and analyzed by flow cytometry using antibodies and methods as described above.

Serum from immune-competent mouse studies was collected from non-heparinized blood collected by RO bleed as described above. Blood was kept at room temperature for 30 min followed by centrifugation at 6000 × *g* for 10 min at 4 °C, then aliquoted to multiple tubes and frozen at −80 °C until used for serum cytokine ELISA or chemistry analyses. Serum chemistry analysis was performed by running samples on a VETSCAN® VS2 Chemistry Analyzer (Zoetis), using the phenobarbital chemistry panel rotor (Zoetis) for ALT and AST quantification as described by the manufacturer’s protocol. Complete blood counts (CBC) analysis on whole blood collected from RO bleed, as described previously, was performed using a VETSCAN® HM5 Hematology Analyzer per the manufacturer’s protocol.

### Immunohistochemistry

Tumor tissue was fixed for up to 3 days in 4% paraformaldehyde (4% PFA, Boston BioProducts) and stored in 70% ethanol until further processing. Immunohistochemistry was performed by the Pathology Core at the City of Hope. Briefly, paraffin-embedded sections (10 µm) were stained with hematoxylin & eosin (H&E, Sigma Aldrich), mouse anti-human CD3 (DAKO), mouse anti-human CD4 (DAKO), mouse anti-human CD8 (DAKO), and mouse anti-human TAG72 (AB16838, Abcam), and rabbit anti-mouse CD3 (AB16669, Abcam). Images were obtained using the Nanozoomer 2.0HT digital slide scanner and the associated NDP.view2 software (Hamamatzu).

### Statistics & Reproducibility

In this study, we evaluated CAR T cells for the treatment of solid tumors using in vitro T-cell functional assays, as well as human xenograft and syngeneic tumor models in mice. We engineered CAR T cells with a membrane-bound IL-12 cytokine and evaluate its therapeutic efficacy in these model systems. All in vitro assays were performed with at least duplicate samples and were repeated in at least two independent experiments. In vivo studies were performed using 6- to 8-week-old NSG or C57BL/6 mice, using at least three mice per group for all in vivo studies to ensure statistical power. Mice were randomized on the basis of tumor volume or bioluminescence imaging to ensure evenly distributed average tumor sizes across each group. In vivo experiments were repeated at least twice. For subcutaneous tumor models, survival was based on the maximum tumor size allowed (about 15 mm in diameter). Data are presented as means ± standard error mean (SEM), unless otherwise stated. Statistical comparisons between groups were performed using the unpaired two-tailed Student’s *t* test to calculate *p* value, unless otherwise stated. GraphPad Prism 8 (GraphPad Software, Inc) was used to generate bar plots and graphs.

### Reporting summary

Further information on research design is available in the Nature Portfolio Reporting Summary linked to this article.

### Supplementary information

Supplementary Information

Reporting Summary

### Source data

Source Data

## Data Availability

The authors declare that all data supporting the findings of the study are available in the article, the Supplementary file, and the Source Data provided with this paper. Source data are provided with this paper.
