# Supplementary material for: Antigen-dependent IL-12 signaling in CAR T cells promotes regional to systemic disease targeting
Source: Nat Commun. 2023 Aug 7;14:4737. doi: 10.1038/s41467-023-40115-1 (PMC10406808; doi:10.1038/s41467-023-40115-1)
Supplement: Supplementary file 1 — Supplementary Information [file 41467_2023_40115_MOESM1_ESM.pdf]

Supplementary Information

Title: Antigen-dependent IL-12 signaling in CAR T cells promotes regional to systemic disease targeting

Author List: Eric Hee Jun Lee[1*], John P. Murad[1*], Lea Christian[1], Jackson Gibson[1], Yukiko Yamaguchi[1], Cody Cullen[1], Diana Gumber[2], Anthony K. Park[1], Cari Young[2], Isabel Monroy[1], Jason Yang[1], Lawrence A. Stern[1], Lauren N. Adkins[1], Gaurav Dhapola[1], Brenna Gittins[1], Wen Chung-Chang[1], Catalina Martinez[3], Yanghee Woo[4], Mihaela Cristea[5], Lorna Rodriguez-Rodriguez[4], Jun Ishihara[6], John K Lee[7], Stephen J. Forman[1,8], Leo D. Wang[8,9], Saul J. Priceman[1,8,#]

Affiliations:

[1]Department of Hematology and Hematopoietic Cell Transplantation, City of Hope, Duarte, CA 91010, USA

[2]Irell and Manella Graduate School of Biological Sciences, Beckman Research Institute of City of Hope, Duarte, CA 91010, USA

[3]Department of Clinical and Translational Project Development, City of Hope, Duarte, CA 91010, USA

[4]Department of Surgery, City of Hope, Duarte, CA 91010, USA

[5]Department of Medical Oncology and Therapeutics Research, City of Hope, Duarte, CA 91010, USA

[6]Department of Bioengineering, Imperial College London, 86 Wood Lane, London W120BZ, UK

[7]Human Biology Division, Fred Hutchinson Cancer Center, Seattle, WA 98019, USA

[8]Department of Immuno-Oncology, Beckman Research Institute of City of Hope, Duarte, CA 91010, USA

[9]Department of Pediatrics, City of Hope, Duarte, CA 91010, USA

*Authors contributed equally

#To whom correspondence may be addressed: Saul J. Priceman, Department of Hematology and Hematopoietic Cell Transplantation, City of Hope, 1500 E. Duarte Rd, Duarte CA 91010, USA. Phone: 626-256-4673; email: spriceman@coh.org.

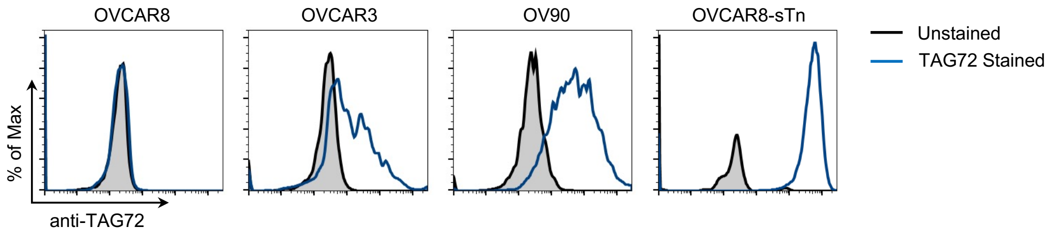

Supplementary Figure 1. TAG72 expression on ovarian tumor cell lines. Flow cytometric analysis of TAG72 expression on human OVCAR8 (TAG72-negative), human OVCAR3 (TAG72-positive), human OV90 (TAG72-positive), human OVCAR8-sTn (TAG72-positive) tumor cell lines.

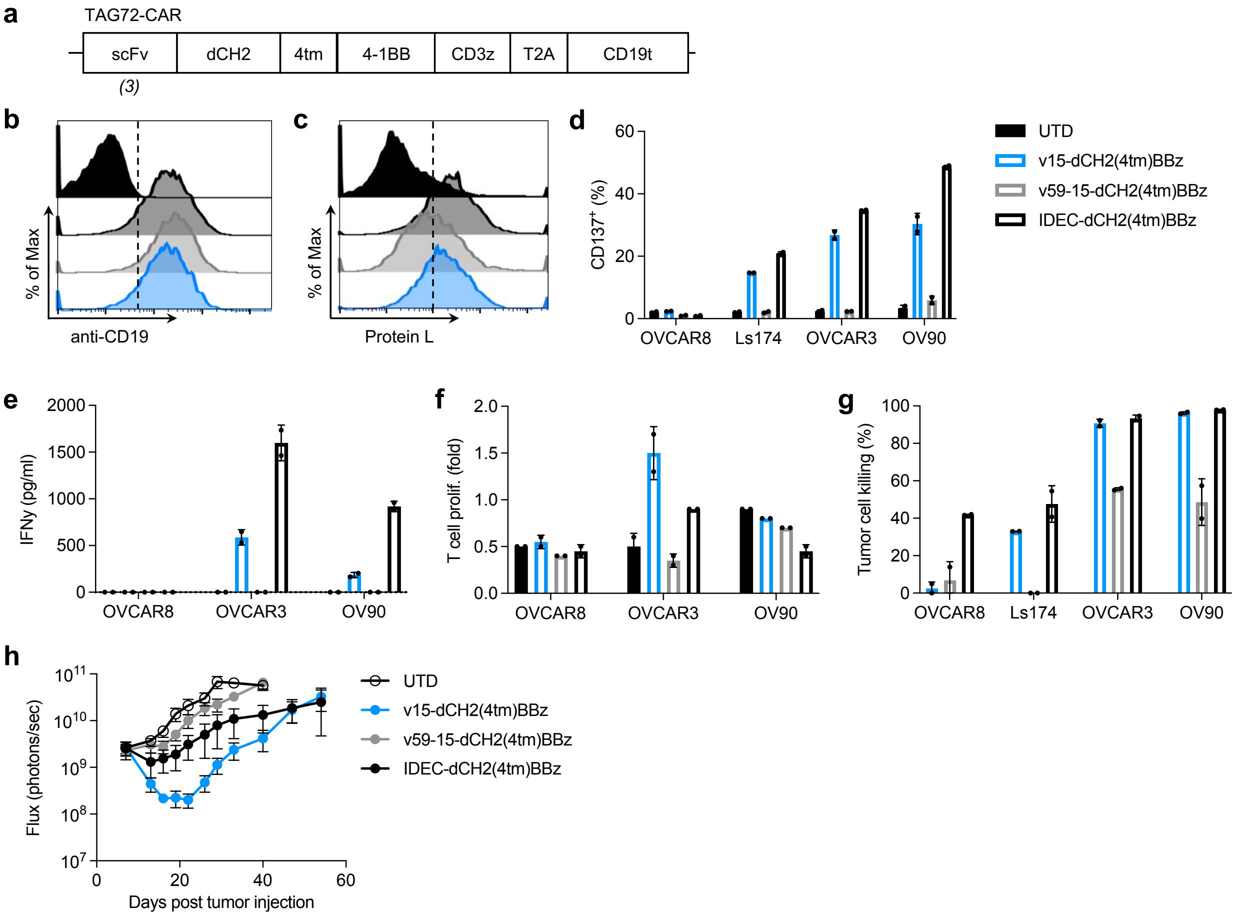

Supplementary Figure 2. *In vitro* and *in vivo* analysis of TAG72-CAR T cells with varying scFv. (a) Diagram of the lentiviral construct with a TAG72-CAR containing three different humanized scFvs (based on CC49 clone) targeting TAG72, with dCH2 extracellular spacer domain (dCH2), CD4 transmembrane (4tm), and intracellular 4-1BB costimulatory domain (BBz) followed by a cytolytic domain (CD3z). A truncated non-signaling CD19 (CD19t), separated from the CAR sequence by a ribosomal skip sequence (T2A), was expressed for identifying lentivirally transduced T cells. **(b-c)** Untransduced (UTD) and three TAG72-CAR T cells positively enriched for CD19t were evaluated by flow cytometry for CD19t expression to detect lentiviral transduction of CARs (b), or Protein L to detect the scFv (c). **(d-g)** Quantification of tumor cell killing **(d)**, IFNγ production **(e)**, T cell proliferation **(f)**, and tumor cell killing **(g)** by the three TAG72-CAR T cells relative to UTD T cells at an E:T ratio of 1:2, following a 24 or 72 hour co-culture with antigen-

positive and -negative tumor targets as described in Methods. n = 2/group, representative of two independent experiments. Data are presented as mean values +/- SD. **(h)** Flux from i.p. OV90(eGFP/ffluc) tumor-bearing mice treated i.p. with UTD or TAG72-CAR T cells. n = 5-6/group. Data are presented as mean values +/- SEM.

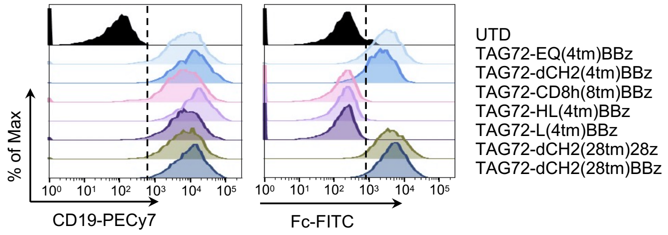

Supplementary Figure 3. TAG72-CAR transduction efficiency. Untransduced (UTD) and 7 different TAG72-CAR T cells positively enriched for CD19t were evaluated by flow cytometry for CD19t expression to detect lentiviral transduction of CARs (left) and fragment constant (Fc) derived spacer containing CARs (right) to support CAR transduction and expression stability data in Figure 1b.

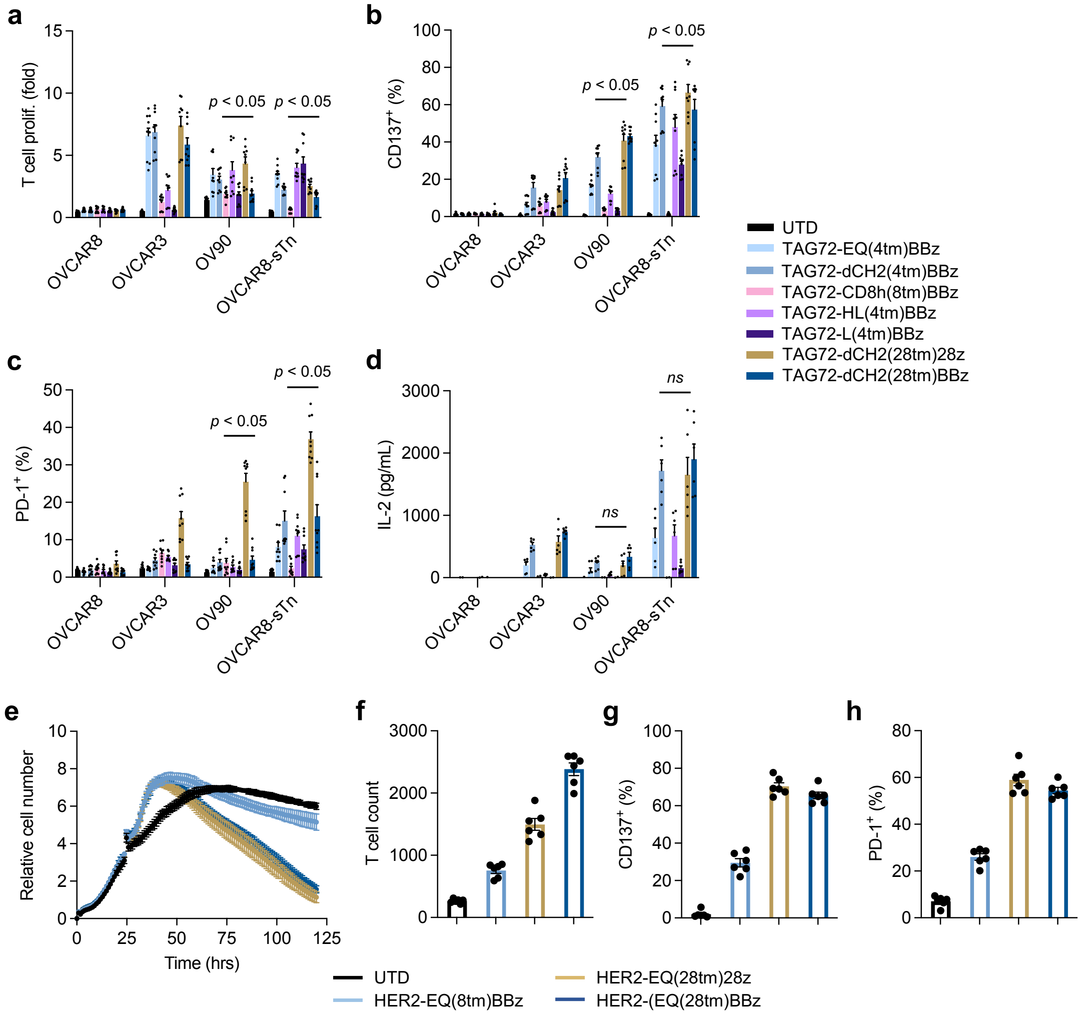

Supplementary Figure 4. *In vitro* analysis of CAR T cells with varying extracellular spacer, transmembrane and costimulatory domains. *In vitro* T cell proliferation in fold change compared to UTD (**a**), expression of CD137 (**b**) and PD-1 (**c**) by flow cytometry, and IL-2 production (**d**) by ELISA (c), of CAR T cells against tumor targets (TAG72- OVCAR8; TAG72+ OVCAR3, OV90, and OVCAR8-sTn) after 24 hr (for ELISA) or 72 hr of co-culture at an effector:target (E:T) ratio of 1:4, to support CAR T cell functional data in Figure 1c-d. n = 9/group from three independent experiments. *P* values indicate differences between TAG72-dCH2(28tm)28z and TAG72-dCH2(28tm)BBz using a two-tailed Student's *t* test. (**e**) HER2-CAR T cell killing of HER2+ SKOV3 cells measured by xCELLigence over 5 days (E:T = 1:20).

Remaining T cells (**f**), along with expression of CD137 (**g**) and PD-1 (**h**) by flow cytometry. n = 6/group. All data are presented as mean values +/- SEM.

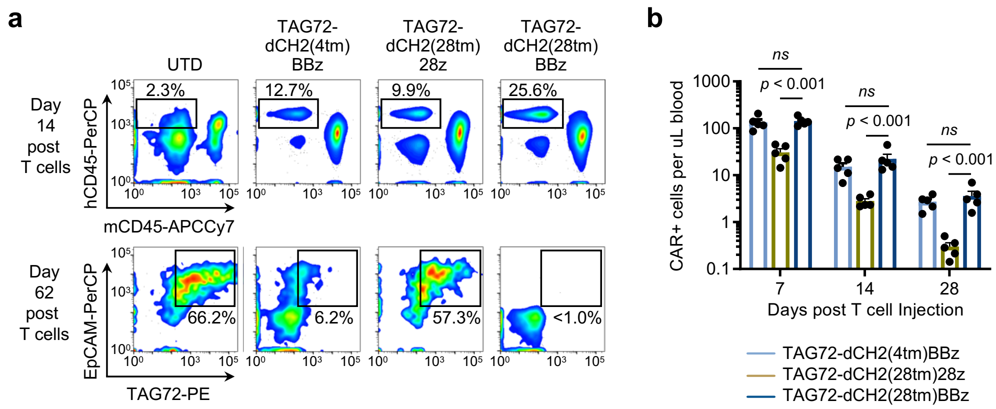

Supplementary Figure 5. TAG72-CAR T cell persistence in peripheral blood. (a) Representative flow cytometric analysis of the frequency of human CD45+ (hCD45) and mouse CD45+ (mCD45) cells in the peritoneal cavity of tumor-bearing mice at day 14 post-treatment (top); human epithelial cell adhesion molecule+ (EpCAM) and tumor associated glycoprotein-72+ (TAG72) tumor cells in the i.p. cavity of tumor-bearing mice at day 62 post-treatment (bottom). **(b)** Quantification of TAG72-CAR T cells per uL of peripheral blood at 7, 14, and 28 days post-treatment. n = 5/group. *P* values indicate differences between TAG72-dCH2(28tm)28z and TAG72-dCH2(28tm)BBz, or between TAG72-dCH2(4tm)BBz and TAG72-dCH2(28tm)BBz, using a two-tailed Student's *t* test. Data are presented as mean values +/- SEM. Extended data from study in Figure 1i-j.

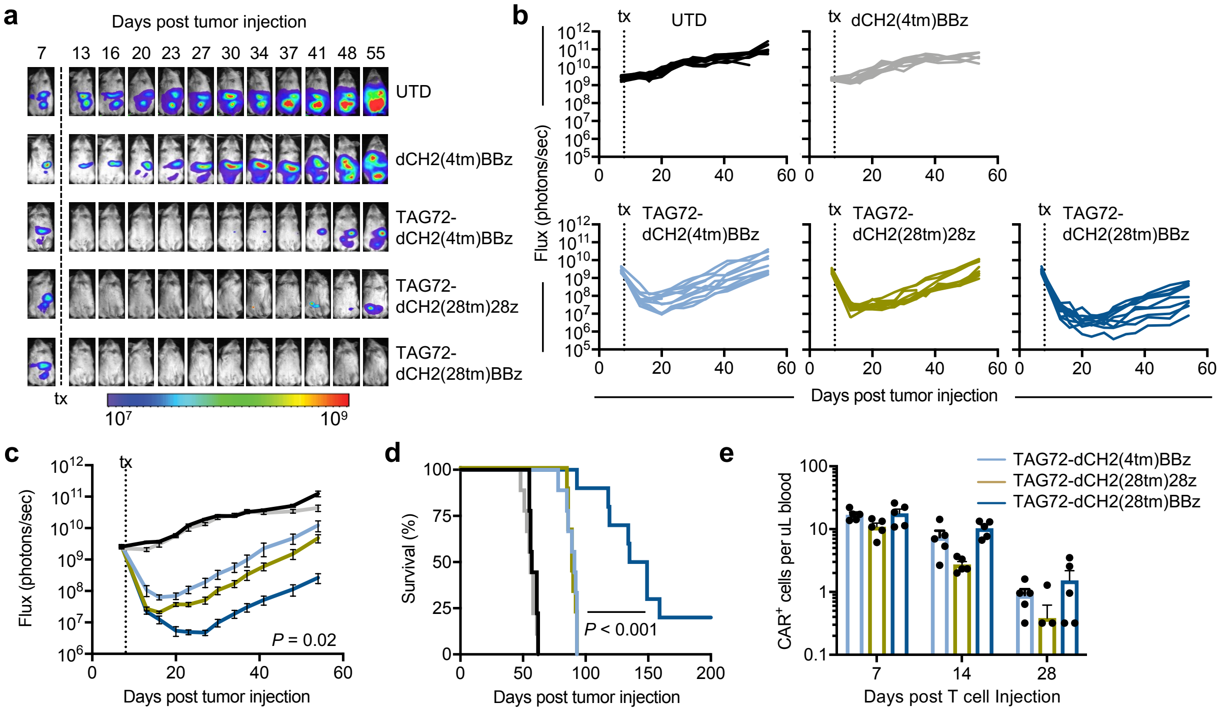

Supplementary Figure 6. Regional intraperitoneal delivery of TAG72-dCH2(28tm)BBz CAR T cells reduces tumor burden and extends overall survival *in vivo*. (a) Representative bioluminescent flux imaging of i.p. OV90 tumor-bearing mice treated i.p. with UTD or TAG72-CAR T cells. **(b-c)** Quantification of flux (b, individual mice per group; c, averages) from i.p. OV90(eGFP/ffluc) tumor-bearing mice treated i.p. with UTD or TAG72-CAR T cells. n = 10-12 mice per group. **(d)** Kaplan-Meier survival for UTD, scFv-less, and TAG72-CAR T cell treated mice. n = 10-12 mice/group. **(e)** Quantification of TAG72-CAR T cells per uL of peripheral blood at 7, 14, and 28 days post-treatment. n = 5/group. All data are presented as mean values +/- SEM. *P* values indicate differences between TAG72-dCH2(28tm)28z and TAG72-dCH2(28tm)BBz using a two-tailed Student's *t* test.

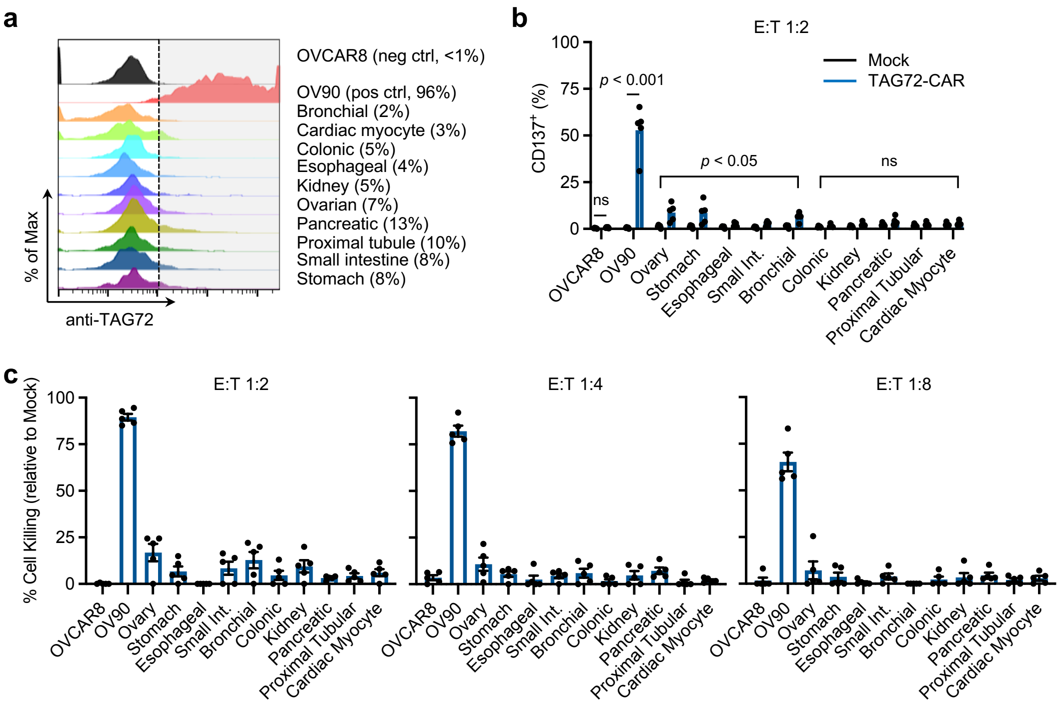

Supplementary Figure 7. *In vitro* safety of TAG72-CAR T cells against normal human cell lines. (a) Flow cytometric analysis of TAG72 expression on the cell surface of TAG72-negative (OVCAR8) tumor cells, TAG72-positive (OV90) tumor cells, and indicated primary human normal cells. **(b-c)** Quantification of CD137 activation (b), and tumor and normal cell killing by TAG72-dCH2(28tm)BBz CAR T cells relative to UTD T cells at varying E:T ratios (c), assessed by flow cytometry following a 48 hour co-culture with indicated cells as described in Methods. All data are from five independent donors, presented as mean values +/- SEM. *P* values in (b) indicate differences between Mock and TAG72-CAR using a two-tailed Student's *t* test.

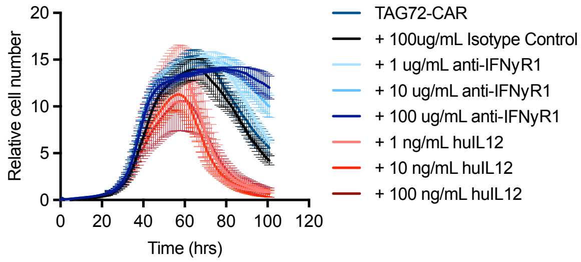

Supplementary Figure 8. TAG72-CAR T cell anti-tumor activity is regulated by IFNγ signaling. Tumor cell killing of OVCAR3 cells by TAG72-CAR T cells (E:T = 1:50) with varying concentrations of anti-IFNγR1 blocking antibody, isotype, and recombinant human IL-12 cytokine by xCELLigence. n = 2 per timepoint. Data are presented as mean values +/- SD.

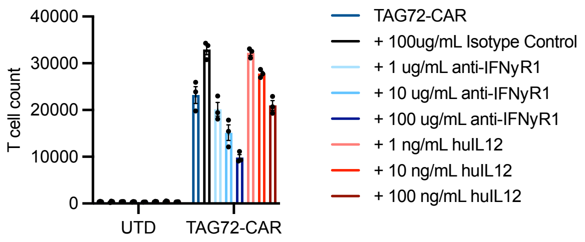

Supplementary Figure 9. T cell counts from assay in Figure 2b. At day 10, remaining T cell counts were analyzed by flow cytometry. n = 3/group. Data are presented as mean values +/- SEM.

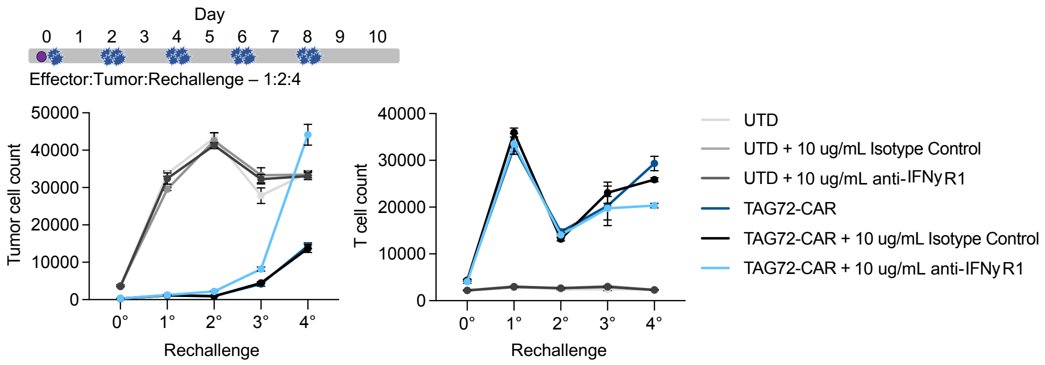

Supplementary Figure 10. Inhibition of IFNγ signaling dampens repetitive tumor cell killing by TAG72-CAR T cells. Schema of repetitive tumor cell challenge assay (top). TAG72-CAR T cells were co-cultured with OV90 cells (E:T = 1:2) and rechallenged with OV90 cells every three days. Remaining viable tumor cells and TAG72-CAR T cells were quantified as described in Methods prior to each tumor cell rechallenge. n = 4/group. Data are presented as mean values +/- SEM.

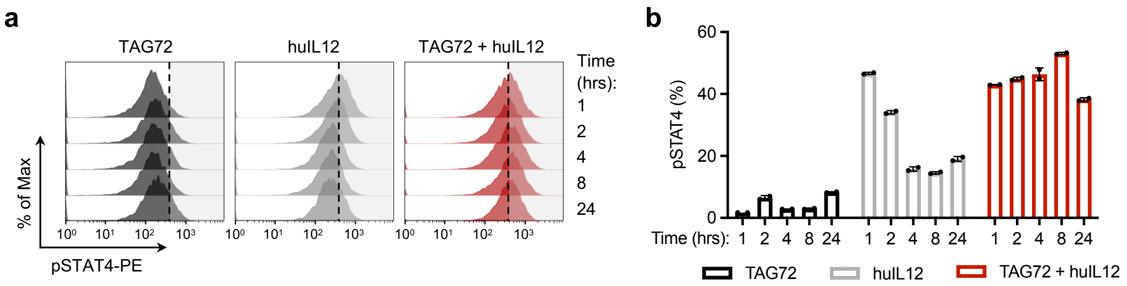

Supplementary Figure 11. Antigen stimulation sustains IL-12 signaling in TAG72-CAR T cells. (a) Representative intracellular flow cytometric analysis of phosphorylated STAT4 (pSTAT4, pY693) in response to TAG72 and/or recombinant huIL12 (10 ng/mL) at indicated timepoints. **(b)** Quantification of pSTAT4 in (a). (b) n = 2/group, representative of two independent experiments. Data are presented as mean values +/- SD.

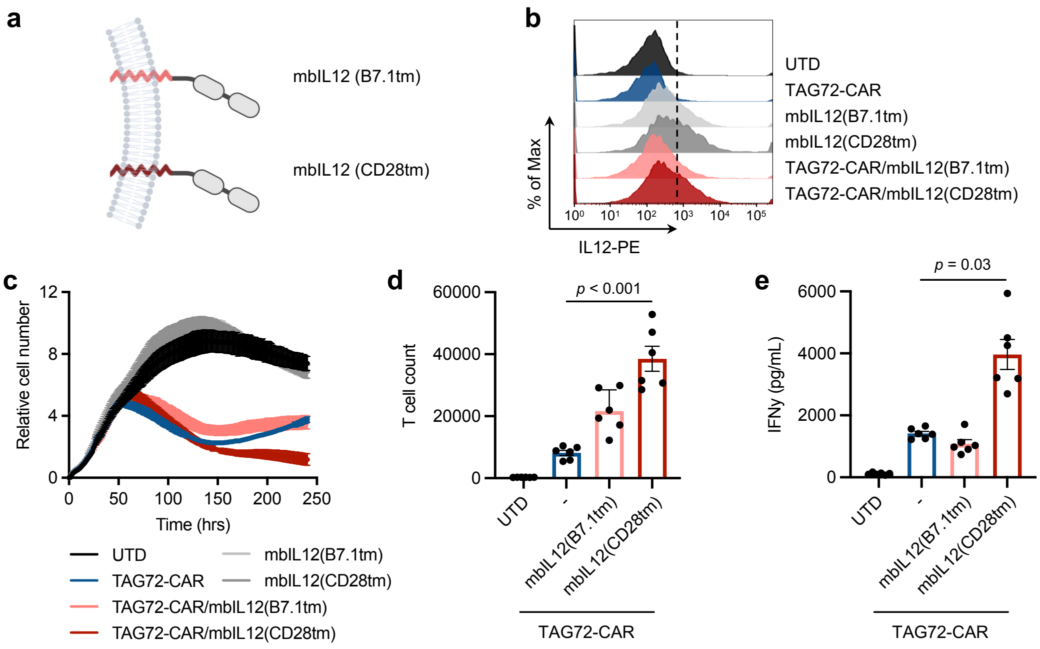

Supplementary Figure 12. Membrane-bound IL-12 construct optimization and *in vitro* mbIL12-engineered CAR T cell activity (a-e) Diagram of the lentiviral construct with two versions of membrane-bound IL-12 (mbIL12) containing B7.1 or CD28 transmembrane domains (**a**). Flow cytometric analysis of IL-12 surface expression on indicated T cells (**b**). TAG72-CAR/mbIL12 T cell killing of OV90 cells (E:T = 1:20) measured by xCELLigence over 10 days (**c**). n = 2/group per timepoint. At day 10, T cell counts were analyzed by flow cytometry (**d**) and IFNγ levels in supernatants were quantified by ELISA (**e**). (c-e) n = 6/group. All data are presented as mean values +/- SEM. *P* values indicate differences between TAG72-CAR (-) and TAG72-CAR/mbIL12(CD28tm) using a two-tailed Student's *t* test.

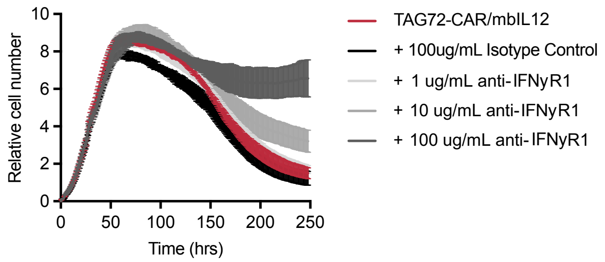

Supplementary Figure 13. IFNγ signaling drives anti-tumor activity in TAG72-CAR/mbIL12 T cells *in vitro.* Tumor cell killing of OV90 cells by TAG72-CAR/mbIL12 T cells (E:T = 1:20) with addition of varying concentrations of anti-IFNγR1 blocking antibody or isotype by xCELLigence over 10 days. n = 2/group per timepoint. Data are presented as mean values +/- SEM.

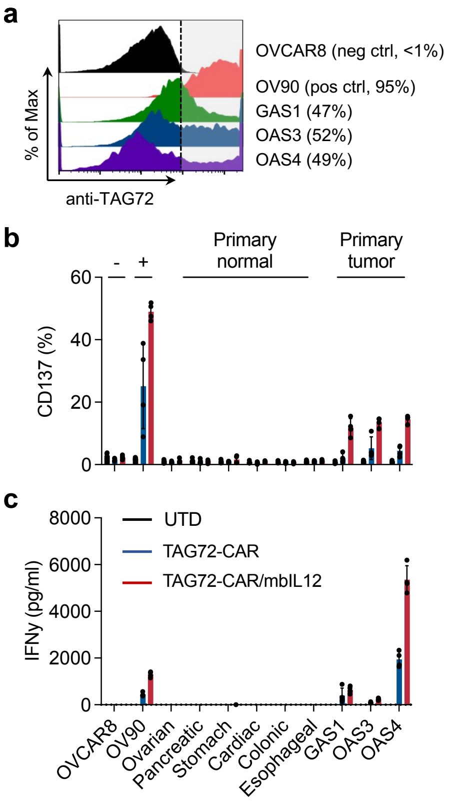

Supplementary Figure 14. *In vitro* safety of TAG72-CAR/mbIL12 T cells against normal human cell lines. (a) Flow cytometric analysis of TAG72 expression on the cell surface of TAG72-negative (OVCAR8) tumor cells, TAG72-positive (OV90) tumor cells, and indicated patient-derived gastric (GAS1) and ovarian (OAS3 and 4) peritoneal ascites. **(b-c)** TAG72-CAR T cells with or without mbIL12 were co-cultured with tumor and normal cell at 1:4 E:T ratio for 48 hours. **(b)** Quantification of CD137 expression in CAR T cells was measured by flow cytometry. **(c)** IFNγ levels in supernatant were quantified by ELISA. (b-c) n = 4/group. All data are presented as mean values +/- SEM.

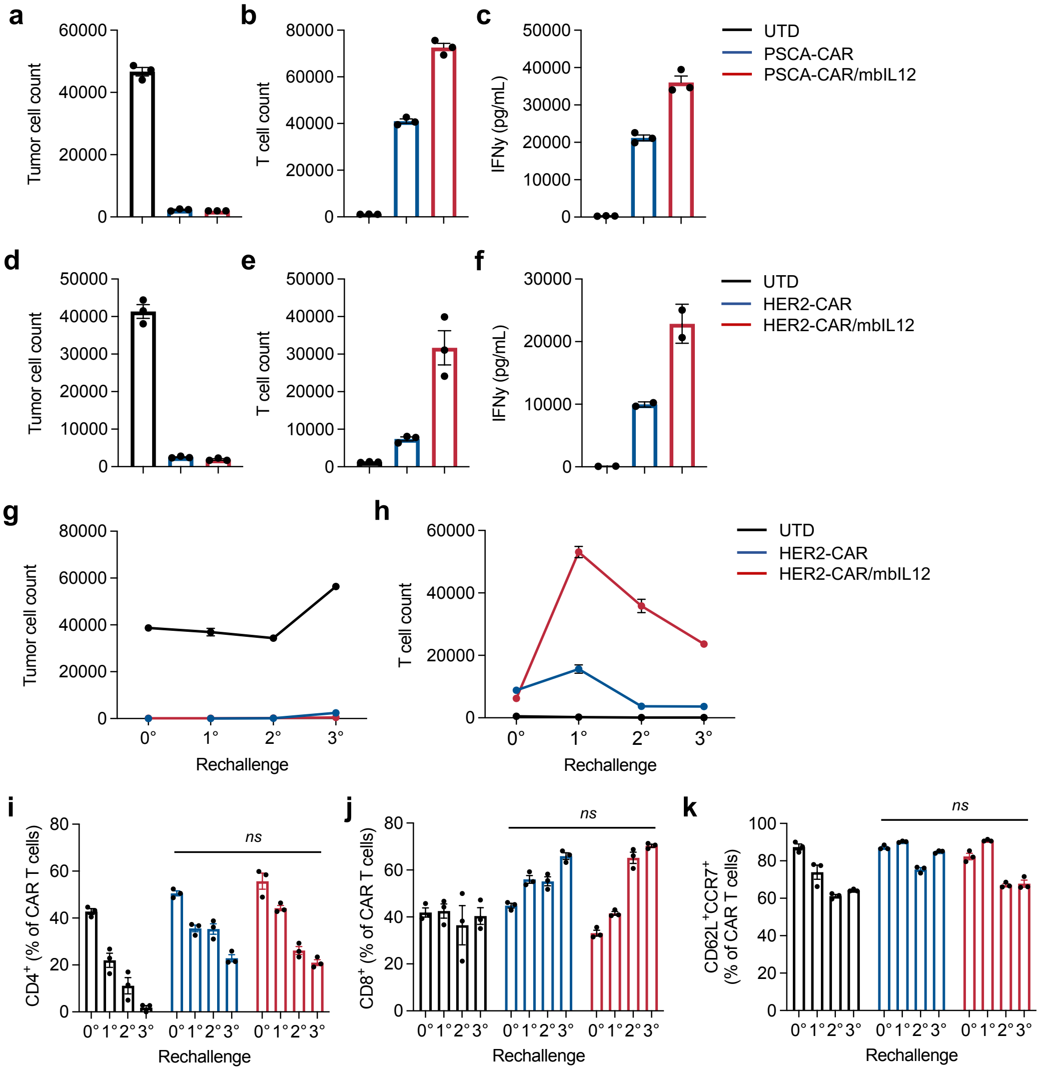

Supplementary Figure 15. mbIL12 signaling improves *in vitro* functionality of PSCA- and HER2-CAR T cells. (a-f) Quantification of tumor cell counts (left), T cell counts (middle), and IFNγ levels in supernatant (right) from PSCA-CAR T cells (**a-c**) and HER2-CAR T cells (**d-f**), following co-culture with antigen-positive targets (PC3-PSCA or 468-HER2) at an E:T ratio of 1:10 for 6 days. (a-e) n = 3/group. Data are presented as mean values +/- SEM. (f) n = 2/group. Data are presented as mean values +/- SD. (**g**) HER2-CAR T cells with or without mbIL12 were co-cultured with 468-HER2 cells (E:T = 1:10) and rechallenged with 468-HER2 cells every three days. Remaining viable tumor cells (**g**) and T cells (**h**) were quantified by flow cytometry prior to every rechallenge and two or three days after the last rechallenge with 468-HER2 cells. Percentage of

(**i-k**) $CD4^+$ (**i**), $CD8^+$ (**j**), and $CD62L^+CCR7^+$ T cells were measured by flow cytometry from the rechallenge assay. (g-j) n = 3/group, representative of two independent experiments. Data are presented as mean values +/- SEM. *P* values indicate differences between HER2-CAR and HER2-CAR/mbIL12 using a paired two-tailed Student's *t* test.

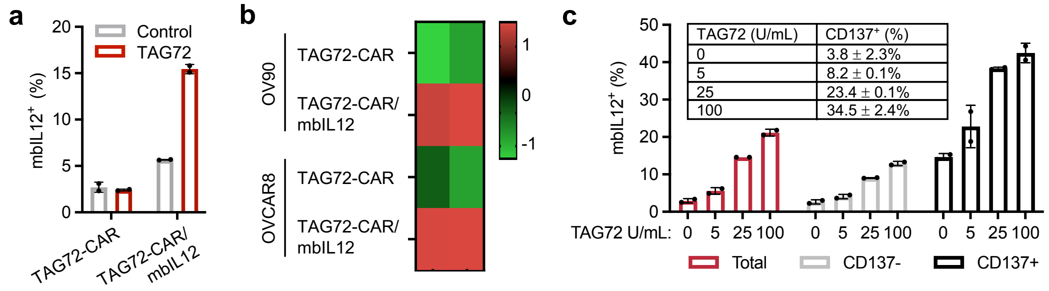

Supplementary Figure 16. Antigen-dependent mbIL12 cell surface expression in CAR T cells. (**a**) Flow cytometric analysis of surface expression of mbIL12 on TAG72-CAR T cells stimulated with plate-bound TAG72 (100 U/mL) or control antigen (PSCA, 2.5 ug/mL). n = 2/group, representative of two independent experiments. Data are presented as mean values +/- SD. (**b**) mRNA levels of IL-12A (left side) and IL-12B (right side) in TAG72-CAR and TAG72-CAR/mbIL12 T cells stimulated with TAG72- OVCAR8 or TAG72+ OV90 tumor cells overnight prior to RNA isolation and bulk RNAseq. (**c**) Flow cytometric analysis of surface expression of mbIL12 on total, or gated on CD137+ or CD137- populations, of TAG72-CAR T cells stimulated with plate-bound TAG72 (100 U/mL). Table inset: CD137+ expression on TAG72-CAR T cells following stimulation with varying concentrations of plate-bound TAG72. n = 2/group. Data are presented as mean values +/- SD.

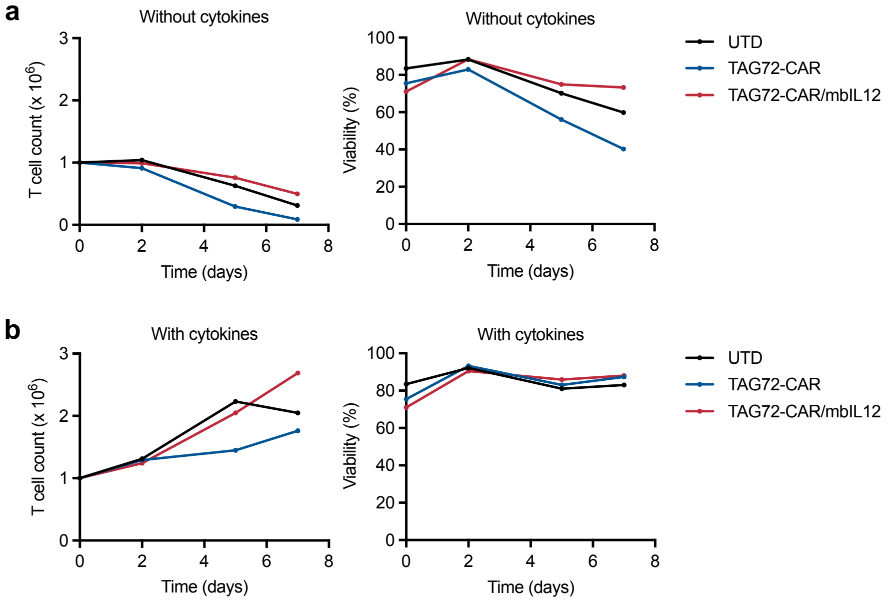

Supplementary Figure 17. mbIL12-engineered TAG72-CAR T cells do not expand and survive in the absence of exogenous cytokines. (a-b) Quantification of T cell count (left) and percentage of viable cells (right) during *ex vivo* culture in the absence (a) or presence (b) of exogenous cytokines as described in Methods. Each sample run in singlicate. Data are representative of two independent experiments.

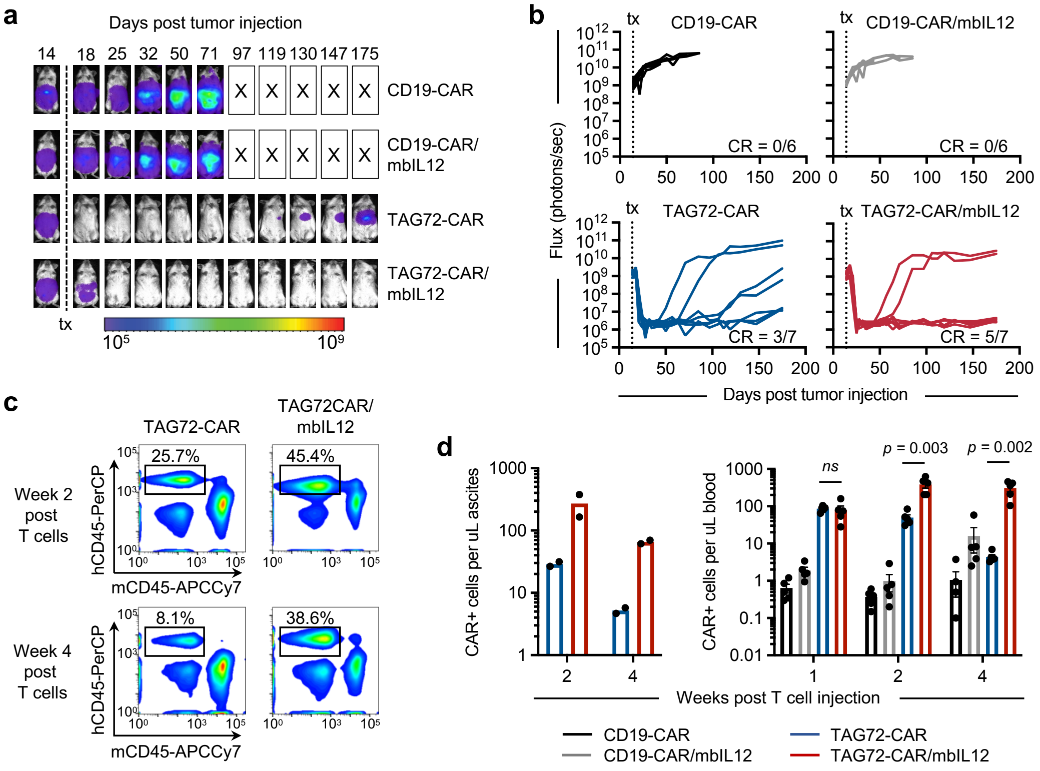

Supplementary Figure 18. Locoregional intraperitoneal delivery of TAG72-CAR/mbIL12 T cells reduces tumor burden and increases regional and systemic CAR T cell persistence *in vivo*. **(a)** Representative bioluminescent flux imaging of i.p. OVCAR3(eGFP/ffluc) tumor-bearing mice treated i.p. with CD19-CAR, CD19-CAR/mbIL12, TAG72-CAR or TAG72-CAR/mbIL12 T cells. **(b)** Quantification of flux (individual mice per group) from mice treated i.p. with CD19-CAR T cells (n = 6/group), CD19-CAR/mbIL12 T cells (n = 6/group), TAG72-CAR T cells (n = 7/group) and TAG72-CAR/mbIL12 T cells (n = 7/group). **(c)** Representative flow cytometric analysis of human CD45+ (hCD45) and mouse CD45+ (mCD45) cells in the peritoneal cavity of tumor-bearing mice at week 2 (top) or week 4 (bottom) post-treatment. **(d)** Quantification of TAG72-CAR T cells per uL of peritoneal ascites (left) at weeks 2 and 4 post-treatment. n = 2 per group. Quantification of TAG72-CAR T cells per uL of peripheral blood (right) at weeks 1, 2, and 4 post-treatment. n = 5/group. Data are presented as mean values +/- SEM. *P* values indicate differences between TAG72-CAR and TAG72-CAR/mbIL12 using a two-tailed Student's *t* test.

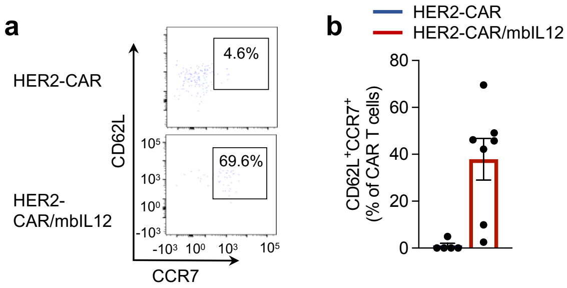

Supplementary Figure 19. Increases in central memory CAR T cells in peripheral blood of HER2-CAR/mbIL12 T cell treated mice. (**a**) Representative flow cytometric analysis of the frequency of HER2-CAR and HER2-CAR/mbIL12 T cells in the peripheral blood from study in Figure 4. (**b**) Quantification of CAR T cells per uL of peripheral blood at 35 days post-treatment. n = 5-7/group. Data are presented as mean values +/- SEM.

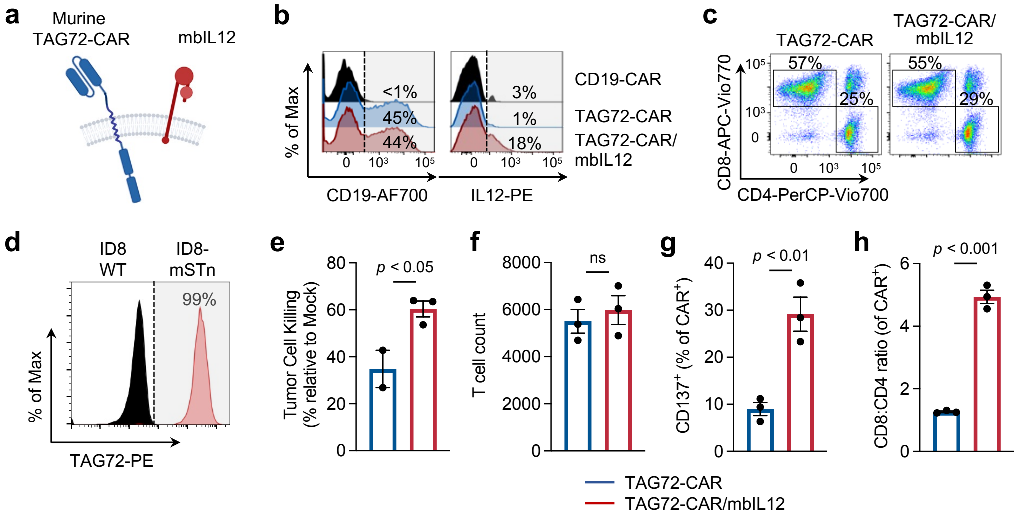

Supplementary Figure 20. Generation and functional characterization *in vitro* of murine TAG72-CAR expressing murine mbIL12. (a) Illustration of dual expression of murine TAG72-CAR and murine mbIL12 on mouse T cell surface. **(b)** Flow cytometric analysis of surface TAG72-CAR expression as detected by expression of truncated murine CD19t (left) and surface murine mbIL12 as detected by anti-mIL12 PE conjugated antibody. **(c)** Flow cytometric analysis of murine CD4 and CD8 ratios in TAG72-CAR and TAG72-CAR/mbIL12. **(d)** Flow cytometric analysis of TAG72 expression in ID8 WT and ID8-mSTn cells. **(e-h)** Functional characterization of 72-hour long term co-culture killing assay tumor killing **(e)** against ID80-mSTn tumor cells at 1:1 E:T **(f)** T cell counts **(g)** CAR T cell CD137 and **(h)** post co-culture CD8 and CD4 ratios in TAG72-CAR vs. TAG72-CAR/mbIL12 T cells. (e-h) n = 2-3/group, representative of two independent experiments. Data are presented as mean values +/- SEM. *P* values indicate differences between TAG72-CAR and TAG72-CAR/mbIL12 using a two-tailed Student's *t* test.

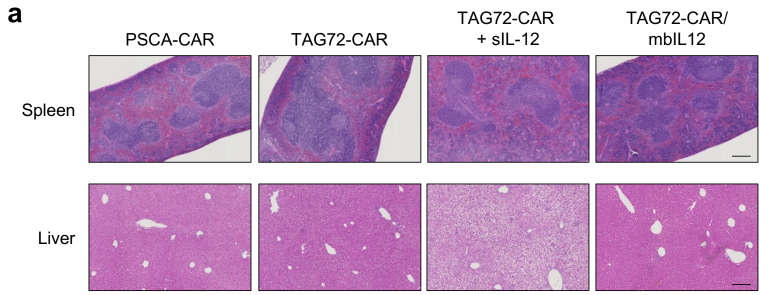

Supplementary Figure 21. Systemic effects of sIL-12 and TAG72-CAR/mbIL12 T cells. H&E of spleen and liver collected from i.p. ID8-mSTn tumor-bearing mice treated with indicated T cells from study in Figure 5. Data are representative of n = 2 mice/group. Scale bar = 200 um.

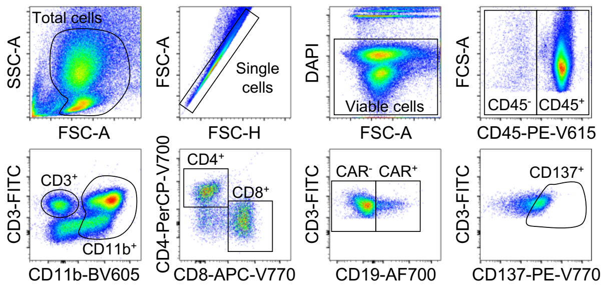

Supplementary Figure 22. Flow cytometry gating strategy for study in Figure 6.

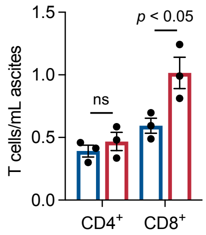

Supplementary Figure 23. Total CD4+ and CD8+ T cells mL of peritoneal ascites from study in Figure 6. n = 3/group. Data are presented as mean values +/- SEM. *P* values indicate differences between TAG72-CAR and TAG72-CAR/mbIL12 using a two-tailed Student's *t* test.

Antibody	Supplier	Catalog #	Clone	Reactivity	Dilution
CD3	BD Biosciences	563109	SK7	anti-human	1:100
CD4	BD Biosciences	340443	SK3	anti-human	1:100
CD8	BD Biosciences	347313	SK1	anti-human	1:100
CD19	BD Biosciences	557835	SJ25C1	anti-human	1:100
CD45	BD Biosciences	555484	2D1	anti-human	1:100
CD69	BD Biosciences	341652	L78	anti-human	1:100
CD137	BD Biosciences	555956	4B4-1	anti-human	1:100
PE-pSTAT3	BD Biosciences	562072	Clone 4	anti-human	1:5
PE-pSTAT4	BD Biosciences	562073	Clone 28	anti-human	1:5
Ep-CAM/CD326	BioLegend	324208	9C4	anti-human	1:100
IL-12(p40/p70)	BD Biosciences	554575	C11.5	anti-human	1:100
CD45	BioLegend	103145	30-F11	anti-mouse	1:100
CD137	Thermofisher	25-1371-82	17B5	anti-mouse	1:100
NK1.1	BioLegend	108733	PK163	anti-mouse	1:100
PD-1	Thermofisher	69-9985-80	J43	anti-mouse	1:100
LAG-3	BioLegend	125227	C9B7W	anti-mouse	1:100
TIM-3	BioLegend	119704	RMT3-23	anti-mouse	1:100
CD11b	BioLegend	101237	M1/70	anti-mouse	1:100
CD44	BioLegend	103010	IM7	anti-mouse	1:100
CD62L	BioLegend	104412	MEL-14	anti-mouse	1:100
CD80	BD Biosciences	740130	16-10A1	anti-mouse	1:100
I-A/I-E (MHC-II)	Thermofisher	64-5321-80	M5/114.15.2	anti-mouse	1:100
CD274 (PD-L1)	BioLegend	124312	10F.962	anti-mouse	1:100
Ly6-C	BioLegend	128029	HK1.4	anti-mouse	1:100
CD11c	BioLegend	117316	N418	anti-mouse	1:100
Ly6-G	Biolegend	127623	1A8	anti-mouse	1:100
CD103	BioLegend	121426	2E7	anti-mouse	1:100
F4/80	BioLegend	123127	BM8	anti-mouse	1:100
IL-12/IL-23 p40	Thermofisher	12-7123-41	17.8	anti-mouse	1:100
TAG72	Novus Biologicals	NBP2-33128	CC49	anti-human/mouse	1:100
biotinylated Protein L	GenScript USA	M00097	N/A	N/A	1:100
Donkey Anti-Rabbit Ig	Invitrogen	A-31573	N/A	anti-rabbit	1:100
Goat Anti-Mouse Ig	BD Biosciences	550589	N/A	anti-mouse	1:100
streptavidin	BD Biosciences	349023	N/A	N/A	1:20
Actin	Cell Signaling	3700	N/A	anti-human	1:2000
p44/42 MAPK (ERK1/2)	Cell Signaling	4695	N/A	anti-human	1:1000
pp44/42 MAPK (pERK1/2)	Cell Signaling	4370	N/A	anti-human	1:1000
SLP76	Cell Signaling	4958	N/A	anti-human	1:1000
pSLP76	Cell Signaling	14745	N/A	anti-human	1:1000
PLCγ1	Cell Signaling	5690	N/A	anti-human	1:1000
pPLCγ1	Cell Signaling	14008	N/A	anti-human	1:1000
anti-rabbit HRP	Cell Signaling	7074	N/A	anti-human	1:1000
anti-mouse HRP	Cell Signaling	7076	N/A	anti-human	1:1000

Supplementary Table 1. Antibody list.
