# Supplementary material for: Antigen-dependent IL-12 signaling in CAR T cells promotes regional to systemic disease targeting
Source: Nat Commun. 2023 Aug 7;14:4737. doi: 10.1038/s41467-023-40115-1 (PMC10406808; doi:10.1038/s41467-023-40115-1)
Supplement: Supplementary file 2 — Reporting Summary [file 41467_2023_40115_MOESM2_ESM.pdf]

nature portfolio

Corresponding author(s): Saul J. Priceman

Last updated by author(s): Jun 26, 2023

Reporting Summary

Nature Portfolio wishes to improve the reproducibility of the work that we publish. This form provides structure for consistency and transparency in reporting. For further information on Nature Portfolio policies, see our Editorial Policies and the Editorial Policy Checklist.

Statistics

For all statistical analyses, confirm that the following items are present in the figure legend, table legend, main text, or Methods section.

n/a	Confirmed	
☐	☒	The exact sample size (*n*) for each experimental group/condition, given as a discrete number and unit of measurement
☐	☒	A statement on whether measurements were taken from distinct samples or whether the same sample was measured repeatedly
☐	☒	The statistical test(s) used AND whether they are one- or two-sided<br>*Only common tests should be described solely by name; describe more complex techniques in the Methods section.*
☒	☐	A description of all covariates tested
☒	☐	A description of any assumptions or corrections, such as tests of normality and adjustment for multiple comparisons
☐	☒	A full description of the statistical parameters including central tendency (e.g. means) or other basic estimates (e.g. regression coefficient) AND variation (e.g. standard deviation) or associated estimates of uncertainty (e.g. confidence intervals)
☐	☒	For null hypothesis testing, the test statistic (e.g. *F*, *t*, *r*) with confidence intervals, effect sizes, degrees of freedom and *P* value noted<br>*Give P values as exact values whenever suitable.*
☒	☐	For Bayesian analysis, information on the choice of priors and Markov chain Monte Carlo settings
☒	☐	For hierarchical and complex designs, identification of the appropriate level for tests and full reporting of outcomes
☒	☐	Estimates of effect sizes (e.g. Cohen's *d*, Pearson's *r*), indicating how they were calculated

Our web collection on statistics for biologists contains articles on many of the points above.

Software and code

Policy information about availability of computer code

Data collection	MACSQuant Analyzer 10 or 16 (Miltenyi Biotec), Cytation 3 (Biotek), xCELLigence (Agilent), Lago X (Spectral Instruments Imaging), ChemiDoc Imaging System (Thermo Scientific)
Data analysis	GraphPad Prism 8 (GraphPad Software, Inc), Flowio v.10 (Flowio LLC), Gen5 (BioTek), Excel (Microsoft), Aura v.4.0 (Spectral Instruments Imaging)

For manuscripts utilizing custom algorithms or software that are central to the research but not yet described in published literature, software must be made available to editors and reviewers. We strongly encourage code deposition in a community repository (e.g. GitHub). See the Nature Portfolio guidelines for submitting code & software for further information.

Data

Policy information about availability of data

All manuscripts must include a data availability statement. This statement should provide the following information, where applicable:

- Accession codes, unique identifiers, or web links for publicly available datasets
- A description of any restrictions on data availability
- For clinical datasets or third party data, please ensure that the statement adheres to our policy

All summary or representative data generated and supporting the findings of this study are available within the paper. Raw data that support the findings are available upon request.

Data analysis section includes these details and complete data availability statements. Source data is also available and will be submitted along with revisions.

Research involving human participants, their data, or biological material

Policy information about studies with human participants or human data. See also policy information about sex, gender (identity/presentation), and sexual orientation and race, ethnicity and racism.

Reporting on sex and gender	Sex and gender were not biased in the studies using human biological materials.
Reporting on race, ethnicity, or other socially relevant groupings	No socially constructed or socially relevant categorization variables were used in the study.
Population characteristics	Leukapheresis products were obtained from consented research participants (healthy donors) under protocols approved by the City of Hope Internal Review Board (IRB)
Recruitment	No specific recruitment were made in these studies.
Ethics oversight	All studies were conducted in accordance with protocols approved by City of Hope's Internal Review Board (IRB)

Note that full information on the approval of the study protocol must also be provided in the manuscript.

Field-specific reporting

Please select the one below that is the best fit for your research. If you are not sure, read the appropriate sections before making your selection.

☒ Life sciences ☐ Behavioural & social sciences ☐ Ecological, evolutionary & environmental sciences

For a reference copy of the document with all sections, see nature.com/documents/nr-reporting-summary-flat.pdf

Life sciences study design

All studies must disclose on these points even when the disclosure is negative.

Sample size	Statistical methods were not used to predetermine the sample size. However, for in vitro experiments, at least duplicates and when available, triplicates, were used for each experiment and were repeated in at least two independent experiments to control for technical replicates. Differences between groups using these samples sizes enabled appropriate statistical analyses. For in vitro experiments using CAR T cells, at least 2 different donors were used to account for biological replicates. For in vivo studies when available at least 7 mice were used per group and repeated in at least 2 independent experiments when applicable for statistical analyses.
Data exclusions	No data were excluded.
Replication	All attempts at replication were successful.<br><br>Studies were replicated at least 2 times.
Randomization	For in vivo studies, mice were randomized on the basis of tumor volume or bioluminescence imaging to ensure evenly distributed average tumor sizes across each group. Randomization was based on plating for all in vitro cell-based assays.
Blinding	Blinding was not relevant to this preclinical study, given bandwidth issues with laboratory staff, and minimization of potential bias based on independent replicates.

Reporting for specific materials, systems and methods

We require information from authors about some types of materials, experimental systems and methods used in many studies. Here, indicate whether each material, system or method listed is relevant to your study. If you are not sure if a list item applies to your research, read the appropriate section before selecting a response.

Materials & experimental systems

n/a	Involved in the study
☐	☒ Antibodies
☐	☒ Eukaryotic cell lines
☒	☐ Palaeontology and archaeology
☐	☒ Animals and other organisms
☒	☐ Clinical data
☒	☐ Dual use research of concern
☒	☐ Plants

Methods

n/a	Involved in the study
☒	☐ ChIP-seq
☐	☒ Flow cytometry
☒	☐ MRI-based neuroimaging

Antibodies

Antibodies used

For extracellular staining, Brilliant Violet 510 (BV510), Brilliant Violet 570 (BV570), Brilliant Violet 605 (BV605), Brilliant Violet 650 (BV650), fluorescein isothiocyanate (FITC), phycoerythrin (PE), peridinin chlorophyll protein complex (PerCP), PerCP-Cy5.5, PECy7, allophycocyanin (APC), or APC-Cy7 (or APC-eFluor780), eFluor506, PE/Dazzle" 594, PerCP-eFluor 710, BD Horizon" Red 718 (R718), Alexa Fluor 488 (AF488), PE-Cy5, -conjugated antibodies were used. Antibodies against CD3 (BD Biosciences, Cat: 563109, Clone: SK7), CD4 (BD Biosciences, Cat: 340443, Clone: SK3), CD8 (BD Biosciences, Cat: 347313, Clone: SK1), CD19 (BD Biosciences, Cat: 557835, Clone: SJ25C1), mouse CD45 (BioLegend, Cat: 103145, Clone: 30-F11), CD45 (BD Biosciences, Cat: 555484, Clone: 2D1), CD69 (BD Biosciences, Cat: 341652, Clone: L78), CD137 (BD Biosciences, Cat: 555956, Clone: 4B4-1), mouse CD137 (Thermofisher, Cat: 25-1371-82, Clone: 17B5), mouse NK1.1 (BioLegend, Cat: 108733, Clone: PK163), mouse PD-1 (Thermofisher, Cat: 69-9985-80, Clone: J43), mouse LAG3 (BioLegend, Cat: 125227, Clone: C9B7W), mouse TIM-3 (BioLegend, Cat: 119704, Clone: RMT3-23), mouse CD11b (BioLegend, Cat: 101237, Clone: M1/70), CD44 (BioLegend, Cat: 103010, Clone: IM7), CD62L (BioLegend, Cat: 104412, Clone: MEL-14), CD80 (BD Biosciences, Cat: 740130, Clone: 16-10A1), mouse I-A/I-E (MHC Class II) (Thermofisher, Cat: 64-5321-80, Clone: M5/114.15.2), mouse CD274 (PD-L1) (BioLegend, Cat: 124312, Clone: 10F.962), Ly6-C (BioLegend, Cat: 128029, Clone: HK1.4), mouse CD11c (BioLegend, Cat: 117316, Clone: N418), mouse Ly-6G (Biolegend, Cat: 127623, Clone: 1A8), mouse CD103 (BioLegend, Cat: 121426, Clone: 2E7), mouse F4/80 (BioLegend, Cat: 123127, Clone: BM8), mouse IL-12/IL-23 p40 (Thermofisher, Cat: 12-7123-41, Clone:17.8), Ep-CAM/CD326 (BioLegend, Clone: 9C4) (Cat: 324208), biotinylated Protein L (GenScript USA, Cat: M00097) (25), TAG72 (Novus Biologicals), Clone, Cat: NBP2-33128, muCC49), Donkey Anti-Rabbit Ig (Invitrogen, Cat: A-31573), Goat Anti-Mouse Ig (BD Biosciences, Cat: 550589), and streptavidin (BD Biosciences, Cat: 349023) were used.For intracellular staining, PE-pSTAT3 (Biolegend, Cat: 562072, pY705) at dilution of 1:5, and PE-pSTAT4 (Biolegend, Cat: 55824, pY693) at a dilution of 1:5 were used. Unless otherwise stated, flow cytometry antibodies were used at dilution of 1:100.

For western blots, Actin (Cell Signaling Technology, CAT#3700, 1:2000), p44/42 MAPK (ERK1/2) (Cell Signaling Technology, Cat: 4695), pp44/42 MAPK (pERK1/2) (Cell Signaling Technology, CAT#4370), SLP76 (Cell Signaling Technology, CAT#4958), pSLP76 (Cell Signaling Technology, CAT#14745), PLCγ1 (Cell Signaling Technology, CAT#5690), pPLCγ1 (Cell Signaling Technology, CAT#14008), Rabbit-HRP (Cell Signaling Technology, CAT#7074) and the Mouse-HRP (Cell Signaling Technology, CAT#7076) were used at 1:1000 for western blots unless othewise noted.

Validation

All antibodies used were validated by titration using known appropriate negative and positive controls, or based on manufacturer recommendations.

Eukaryotic cell lines

Policy information about cell lines and Sex and Gender in Research

Cell line source(s)

OV90 was purchased from ATCC (ATCC-CRL-11732).
OVCAR3 was purchased from ATCC (ATCC HTB-161).
OVCAR8 was obtained from Dr. Carlotta Glackin (COH).
HT1080 was purchased from ATCC (ATCC CCL-121).
DU145 was purchased from ATCC (ATCC HTB-81).
293T was purchased from ATCC (ATCC CRL-3216).
SKBR3 was purchased from ATCC (ATCC-HTB-30).
MDA-MB-468 was purchased from ATCC (ATCC HTB-132).
BBM1 was obtained from Dr. Rahul Jandial (COH) (Neman et al. PNAS 2014, https://doi.org/10.1073/pnas.13220981).
ID8 was obtained from Dr. Karen Aboody (COH).
Human Primary Colonic Epithelial Cells was obtained from Cell Biologics (H-6047).
Human Primary Esophageal Epithelial Cells was obtained from Cell Biologics (H-6046).
Human Primary Kidney Epithelial Cells was obtained from Cell Biologics (H-6034).
Human Primary Ovarian Epithelial Cells was obtained from Cell Biologics (H-6036).
Human Primary Pancreatic Epithelial Cells was obtained from Cell Biologics (H-6037).
Human Primary Proximal Tubular Epithelial Cells was obtained from Cell Biologics (H-6015).
Human Primary Small Intestine Epithelial Cells was obtained from Cell Biologics (H-6051).
Human Primary Stomach Epithelial Cells was obtained from Cell Biologics (H-6039).
Human Cardiac Myocytes was obtained from PromoCell (C-12810).
Human Bronchial Epithelial Cells was obtained from Lonza (CC-2541).

Authentication

The cell lines that were directly purchased from ATCC, PromoCell, and Lonza were authenticated per manufacturer policies. ATCC, PromoCell, and Lonza use morphology, karyotyping, and PCR based approaches to confirm the identity of human cell lines and to rule out both intra- and inter-species contamination. These include an assay to detect species specific variants of

	the cytochrome C oxidase I gene (COI analysis) to rule out inter-species contamination and short tandem repeat (STR) profiling to distinguish between individual human cell lines and rule out intra-species contamination.
Mycoplasma contamination	The cell lines used were tested for the presence of mycoplasma contamination using MycoAlert Mycoplasma Detection Kit (Lonza), which was determined to be negative.
Commonly misidentified lines (See ICLAC register)	No commonly misidentified lines were used.

Animals and other research organisms

Policy information about studies involving animals; ARRIVE guidelines recommended for reporting animal research, and Sex and Gender in Research

Laboratory animals	NOD-SCID-Gamma chain deficient female mice and C57BL/6J female mice approximately 6-8 weeks were used.<br><br>Mice are housed with 12 hours of light and 12 hours of darkness. Temperature Range: 68F to 75F<br>Humidity: 30% to 70%.
Wild animals	No wild animals were used.
Reporting on sex	For studies which were specific to our ovarian cancer models, we used female mice. In the breast cancer models, we also used female mice but acknowledge this disease is not limited to females. No sex-based analyses were performed in the study for these reasons.
Field-collected samples	No field-collected samples were used.
Ethics oversight	All studies were conducted in accordance with protocols approved by City of Hope’s Internal Review Board (IRB) and Institutional Animal Care and Use Committee (IACUC). All relevant animal use guidelines and ethical regulations were followed.

Note that full information on the approval of the study protocol must also be provided in the manuscript.

Flow Cytometry

Plots

Confirm that:

☒ The axis labels state the marker and fluorochrome used (e.g. CD4-FITC).

☒ The axis scales are clearly visible. Include numbers along axes only for bottom left plot of group (a 'group' is an analysis of identical markers).

☒ All plots are contour plots with outliers or pseudocolor plots.

☒ A numerical value for number of cells or percentage (with statistics) is provided.

Methodology

Sample preparation	For flow cytometric analysis, cells were resuspended in FACS buffer (Hank’s balanced salt solution without Ca2+, Mg2+, or phenol red (HBSS–/–, Life Technologies) containing 2% FBS and 1 × AA). Cells were incubated with primary antibodies for 30 min at 4°C in the dark. For secondary staining, cells were washed twice prior to 30 min incubation at 4°C in the dark with either Brilliant Violet 510 (BV510), Brilliant Violet 570 (BV570), Brilliant Violet 605 (BV605), Brilliant Violet 650 (BV650), fluorescein isothiocyanate (FITC), phycoerythrin (PE), peridinin chlorophyll protein complex (PerCP), PerCP-Cy5.5, PECy7, allophycocyanin (APC), or APC-Cy7 (or APC-eFluor780), eFluor506, PE/Dazzle" 594, PerCP-eFluor 710, BD Horizon" Red 718 (R718), Alexa Fluor 488 (AF488), PE-Cy5, -conjugated antibodies were used. Antibodies against CD3 (BD Biosciences, Cat: 563109, Clone: SK7), CD4 (BD Biosciences, Cat: 340443, Clone: SK3), CD8 (BD Biosciences, Cat: 347313, Clone: SK1), CD19 (BD Biosciences, Cat: 557835, Clone: SJ25C1), mouse CD45 (BioLegend, Cat: 103145, Clone: 30-F11), CD45 (BD Biosciences, Cat: 555484, Clone: 2D1), CD69 (BD Biosciences, Cat: 341652, Clone: L78), CD137 (BD Biosciences, Cat: 555956, Clone: 4B4-1), mouse CD137 (Thermofisher, Cat: 25-1371-82, Clone: 17B5), mouse NK1.1 (BioLegend, Cat: 108733, Clone: PK163), mouse PD-1 (Thermofisher, Cat: 69-9985-80, Clone: J43), mouse LAG3 (BioLegend, Cat: 125227, Clone: C9B7W), mouse TIM-3 (BioLegend, Cat: 119704, Clone: RMT3-23), mouse CD11b (BioLegend, Cat: 101237, Clone: M1/70), CD44 (BioLegend, Cat: 103010, Clone: IM7), CD62L (BioLegend, Cat: 104412, Clone: MEL-14), CD80 (BD Biosciences, Cat: 740130, Clone: 16-10A1), mouse I-A/I-E (MHC Class II) (Thermofisher, Cat: 64-5321-80, Clone: M5/114.15.2), mouse CD274 (PD-L1) (BioLegend, Cat: 124312, Clone: 10F.962), Ly6-C (BioLegend, Cat: 128029, Clone: HK1.4), mouse CD11c (BioLegend, Cat: 117316, Clone: N418), mouse Ly-6G (Biolegend, Cat: 127623, Clone: 1A8), mouse CD103 (BioLegend, Cat: 121426, Clone: 2E7), mouse F4/80 (BioLegend, Cat: 123127, Clone: BM8), mouse IL-12/IL-23 p40 (Thermofisher, Cat: 12-7123-41, Clone:17.8), Ep-CAM/CD326 (BioLegend, Clone: 9C4) (Cat: 324208), biotinylated Protein L (GenScript USA, Cat: M00097) (25), TAG72 (Novus Biologicals), Clone, Cat: NBP2-33128, muCC49), Donkey Anti-Rabbit Ig (Invitrogen, Cat: A-31573), Goat Anti-Mouse Ig (BD Biosciences, Cat: 550589), and streptavidin (BD Biosciences, Cat: 349023, 1:20 dilution) were used. Cell viability was determined using 4′, 6-diamidino-2-phenylindole (DAPI, Sigma, Cat: D8417). Unless otherwise stated, antibodies were used at dilution of 1:100. Flow cytometry was performed on a MACSQuant Analyzer 10 or MACSQuant Analyzer 16 (Miltenyi Biotec), and the data was analyzed with FlowJo software (v10.8.1, TreeStar).

For intracellular flow cytometry, CAR T cells were thawed and rested in IL-2 (50 U/mL) & IL-15 (0.5 ng/mL) overnight at 1 x 106 cells/mL. On the following day, CAR T cells were washed twice in 1x PBS and suspended at 1 x 106 cells/mL in media without serum or cytokines. 1 x 105 cells were plated per well in a 96-well plate to rest overnight. The next day, cells were stimulated with either soluble cytokine [IL-2 (50 U/mL), IL-15 (0.5 ng/mL), IL-12 (10 ng/mL)] or transferred to a high-binding 96-well plate pre-coated with indicated amounts of control or TAG72 antigen (BioRad). Reagents and buffers for flow cytometry processing were pre-chilled on ice unless otherwise stated. Following antigen stimulation, cells were washed with FACS buffer (supplemented with 0.1% sodium azide) and then fixed in pre-warmed 1x BD Phosflow Lyse/Fix buffer (558049) at 37oC for 10 minutes. Cells were then washed with FACS buffer and if required, stained with the extracellular antibodies on ice for 30 minutes in the dark. Stained cells were washed and suspended in pre-chilled (-20oC) BD Perm Buffer III (558050) and kept on ice for 30 minutes. Following a wash, cells were suspended in human FC block (Miltenyi Biotec Inc., FLP3330, 1:50) and kept on ice for 30 minutes, washed and stained at dilution of 1:5 with intracellular antibodies: PE-pSTAT3 (Biolegend, Cat: 562072, pY705) and PE-pSTAT4 (Biolegend, Cat: 55824, pY693). Data was acquired on a MACSQuant Analyzer 16 cytometer (Miltenyi) and analyzed with FlowJo v10.8.

Instrument

All flow cytometry was performed on MACSQuant Analyzer 10 or MACSQuant Analyzer 16 (Miltenyi Biotec)

Software

Flow cytometry data (FCS files) were analyzed using FlowJo v10 (TreeStar)

Cell population abundance

At least 10,000 events were collected for each flow cytometry sample

Gating strategy

Relevant populations were gated as follows: Live DAPI- cells --> gating of interest (i.e. CD45+ for T cells and CD45- for tumors). Negative and positive controls were included in each assay, when available. Refer to supplementary figure 22 for representative gating strategy example.

☒ Tick this box to confirm that a figure exemplifying the gating strategy is provided in the Supplementary Information.
